# Stabilisation of half MCM ring by Cdt1 during DNA insertion

**DOI:** 10.1038/s41467-021-21932-8

**Published:** 2021-03-19

**Authors:** Marina Guerrero-Puigdevall, Narcis Fernandez-Fuentes, Jordi Frigola

**Affiliations:** 1 Institut d’Investigació Biomèdica de Girona Dr. Josep Trueta (IDIBGI), Parc Hospitalari Martí i Julià de Salt, Catalunya, Spain; 2 Department of Biosciences, U Science Tech, Universitat de Vic-Universitat Central de Catalunya, Vic, Spain

**Keywords:** DNA synthesis, DNA synthesis, Origin firing

## Abstract

Origin licensing ensures precise once per cell cycle replication in eukaryotic cells. The Origin Recognition Complex, Cdc6 and Cdt1 load Mcm2-7 helicase (MCM) into a double hexamer, bound around duplex DNA. The complex formed by ORC-Cdc6 bound to duplex DNA (OC) recruits the MCM-Cdt1 complex into the replication origins. Through the stacking of both complexes, the duplex DNA is inserted inside the helicase by an unknown mechanism. In this paper we show that the DNA insertion comes with a topological problem in the stacking of OC with MCM-Cdt1. Unless an essential, conserved C terminal winged helix domain (C-WHD) of Cdt1 is present, the MCM splits into two halves. The binding of this domain with the essential C-WHD of Mcm6, allows the latching between the MCM-Cdt1 and OC, through a conserved Orc5 AAA-lid interaction. Our work provides new insights into how DNA is inserted into the eukaryotic replicative helicase, through a series of synchronized events.

## Abstract (web summary)

During pre-Replication Complex, eukaryotic cells load two MCMs into a head-to-head Double Hexamer around duplex DNA (DH). Here the authors preRC assembly assay with purified proteins to reveal insights into S. cerevisiae’s first steps that lead to the DH formation.

## Introduction

During origin licensing, eukaryotic cells load two MCMs into a head-to-head double hexamer around duplex DNA (DH)^1,2^. This process, also known as preRC assembly (from pre-replication complex), is the basis of chromosome replication regulation. DH is the only known replisome precursor. This can only be assembled in the G1 phase, thus origin licensing has a profound effect in the S phase, when chromosome replication takes place.

Origin licensing starts with the origin recognition complex (ORC) binding to origin DNA in its ATP-bound form. Subsequently, an ORC-related member, Cdc6, binds to the ORC in its own ATP-bound form. It is this ORC-Cdc6 complex, bound to double-strand DNA (dsDNA) and ATP (hereafter, OC), that further recruits the MCMs to proceed with the DH formation^3^. Consequently, most of the preRC members are already bound to origin DNA before MCM recruitment starts. In fact, the only preRC member that is not bound to DNA is Cdt1, which forms a stable complex with MCM in solution, named MCM-Cdt1. Therefore, it is the MCM-Cdt1 which, when recruited by the OC, will proceed with the helicase loading and lead to the DH formation.

Cdt1 is the most regulated preRC member in human cells. It is an essential origin licensing protein that is conserved in all eukaryotic cells. Its degradation in the S phase is mediated by two independent E3 ubiquitin ligase complexes, CRL1^skp2^ and CRL4^Cdt2^. Geminin, a substrate of the anaphase-promoting complex/cyclosome, and the only extra preRC component in metazoans, regulates its function in G2/M. In addition, Cdt1 can be phosphorylated at multiple sites along the cell cycle, in response to different cellular pathways^4^. The tight regulation of Cdt1 indicates its prominent role in genome duplication. In fact, an increase of Cdt1 levels is enough to induce re-replication and genome instability in human cells^5,6^. Accordingly, Cdt1 transcription is driven by the E2F and Rb-E2F pathway, which has been found to be one of the most mutated pathways in a variety of cancers^7^. Understanding Cdt1 function is critical in dissecting its role in cancer transformation and/or progression. The implications of consequent findings, dependent on this further understanding, are considerable, as it is expected that this will ultimately lead to the development of new therapeutic treatments.

The development of a preRC assembly assay with purified proteins has been a leap forward in the replication field. Not only has this led to the discovery of DH^1,2^ but also to the unveiling of crucial steps in the DH formation^8–11^. Because it is an ATP-dependent process, if ATP hydrolysis is not allowed in the reaction (also known as the recruitment), all preRC members remain bound to the origin DNA. Further characterization by cryogenic electron microscopy (cryo-EM) led to the structural characterization of this recruited complex, named OCCM (from ORC-Cdc6-Cdt1-MCM)^8^. Multiple lines of evidence strongly suggest that it is a bona fide intermediate of the loading reaction. It has been shown that ORC and Cdc6 ATP hydrolysis are not required for DH formation^12,13^ and Cdt1 inhibits MCM ATPase activity^14,15^. Furthermore, using time-resolved EM, it has been recently observed that the OCCM disappears as DHs are assembled^10^. Finally, near atomic resolution of the OCCM has shown that both complexes, OC and MCM-Cdt1, adopt a ring conformation, stacked on one another, with dsDNA running through their central cavities^8^. On the other hand, if ATP hydrolysis is allowed, the MCM-Cdt1 complexes have two different outcomes. If all preRC members are present and coupled with the right posttranslational modification, they will form DHs (loading). However, if the criteria for correct licensing are not met, they will be irreversibly dissociated from origin DNA, by an ATPase-dependent quality control mechanism (QC or release)^16^.

Arguably one of the key events during origin licensing is the dsDNA incorporation by MCM. In solution, the MCM can coexist with or without Cdt1. We and others have shown that Cdt1 binds the half MCM hexamer, composed by Mcm2/6/4^16,17^. Recently, based on biochemical and single-particle EM, we have proposed that Cdt1 binding stabilizes MCM in a left-handed spiral open at the Mcm2-5 gate^15^. Unfortunately, the resolution was insufficient to determine whether the opening was wide enough to incorporate dsDNA or not. With regards to this, a cryo-EM study has suggested that the opening at the Mcm2-5 gate is not wide enough to accommodate dsDNA^17^. Additionally, and in support of the aforementioned findings, there has, as yet, been no evidence of DNA-binding activity by MCM-Cdt1. Thus, it is possible that an additional conformational change, widening the gate, will have to occur to incorporate duplex DNA. Essentially, an important question still remains unanswered, regarding the way in which the dsDNA is incorporated by the MCM-Cdt1 complex in its central channel.

Here we have used the reconstituted preRC assembly assay to further understand this matter. We have used a reconstituted preRC assay with purified proteins to dissect the first steps that lead to the DH formation, together with a systematic study of the C-terminal winged helix domain (C-WHD) of all Mcm subunits. We have found that the MCM hexamer exhibits a high degree of dynamism during helicase recruitment and loading. The half hexamer, composed by Mcm3/5/7, shows great stability and contacts the OC complex in a sequenced manner, through the C-WHDs of Mcm3 and 7, regardless of the presence of Cdt1. However, the other half hexamer, Mcm2/6/4, can be neither efficiently recruited nor loaded in the absence of Cdt1. Specifically, it requires its extended C-WHD. The interaction of this domain with the Mcm6 C-WHD allows the latching between both complexes, MCM-Cdt1 and OC, via a conserved Orc5 interaction. Overall, we are proposing a model detailing how a series of sequenced events, by the C-WHDs of the MCM and Cdt1, allow the insertion of dsDNA inside the core of the replicative helicase of *Saccharomyces cerevisiae*.

## Results

### Mcm7 C-WHD is essential for MCM-Cdt1 recruitment

**Fig. 1:**
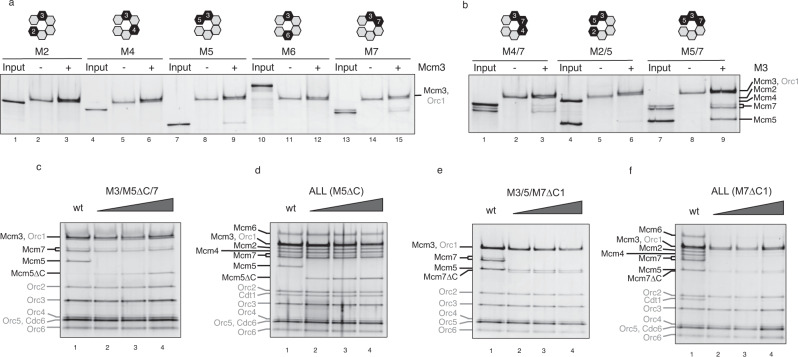
Mcm7 C-WHD is required for MCM-Cdt1 recruitment. **a** The recruitment of the individual Mcm subunits (M2, 4, 5, 6 and 7) by the OC complex (ORC and Cdc6 bound to a PCR product containing ARS305 and 1049 bp long) was followed using recruitment conditions (ATPγS and low salt washes), under the presence (+) or absence (−) of Mcm3. 20% of each Mcm subunit tested is shown in the input lane. Relative position in the Mcm2-7 ring, of Mcm3 and the subunit tested, are highlighted in black at the top of the figure. Note that the Orc1 subunit and Mcm3 run in similar positions in the gel. **b** Neighbouring dimers of Mcm3 are tested for recruitment, as in **a**. **c** Recruitment of the trimer, Mcm5/3/7, using either Mcm5wt (wt) or increasing amounts of an Mcm5 containing a deletion of the C terminus winged helix domain (C-WHD, M5∆C). Loading factors are labelled in grey and Mcm2-7 in black. **d** Recruitment of the heptameric MCM-Cdt1 complex containing either Mcm5 wt (lane 1) or with increasing amounts of M5∆C (lanes 2–4). **e** Recruitment of the trimer Mcm5/3/7, with either Mcm7 wt (lane 1) or increasing amounts of an Mcm7 protein that lacks its C-WHD (M7∆C1, lanes 2–4). **f** Recruitment of the MCM-Cdt1 with either Mcm7 wt (lane 1) or with increasing amounts of M7∆C1 (lanes 2–4). See also Supplementary Fig. 2c–e.

It has been shown that Mcm3 C-WHD plays a critical role in the initiation of MCM recruitment during preRC assembly^9,11,16,18^. Remarkably, the data suggest that, without this domain, none of the different MCM-Cdt1 subunits can be recruited by the OC. Even more interestingly, a single mutation on the last amino acid of this domain completely abolishes the recruitment in vitro, and in vivo this mutation is unable to complement the suppression of Mcm3 wild type (wt)^16^. These results strongly suggest that the Mcm3 subunit is the first Mcm to contact the OC. Therefore, we chose to investigate which other subunits could be recruited using the preRC assay with purified proteins. It is known that the use of a non-chromatinized DNA template establishes the licensing reaction as a non-ARS sequence specific^1,2,10,11^. Therefore, we tested the specificity of the recruitment of the individual Mcm subunits, in a Mcm3-dependent manner, as we previously published^16^. Only the neighbouring subunits of Mcm3, 5 and 7 can be recruited partially (Fig. 1a). As we have shown previously, Cdt1 cannot be recruited together with Mcm3 (Supplementary Fig. 2c). Due to the fact that neighbouring subunits could be affecting these minimal interactions, we tested the binding of the dimers around Mcm3 (Fig. 1b). While Mcm2 could not be recruited along with the dimer Mcm3-5 (lane 6), as predicted by the presence of the Mcm2-5 gate^19,20^, we observed a partial recruitment of Mcm4 by the dimer Mcm3-7 (lane 3). However, only stoichiometric amounts of half hexamer Mcm5/3/7 could be recruited (lane 9). It is noteworthy that all these subcomplexes are released from DNA if we allow ATP hydrolysis (Supplementary Fig. 2d), as expected by the QC. These results suggest a dynamic MCM complex, highlighting the great stability of the half hexamer Mcm5/3/7, upon helicase recruitment by the OC.

All the MCM subunits, with the exception of Mcm2 in *S. cerevisiae*, contain a C-WHD. Structural data have suggested that these long and flexible domains may play an important role during the recruitment and loading of the MCM^21^. However, biochemical data supporting this hypothesis is still limited. Here we have investigated the roles of Mcm5 and 7 C-WHDs. We observed that Mcm5 C-WHD is required to some degree for recruitment of the trimer 5/3/7 (Fig. 1c), although higher amounts almost equal the recruitment by the Mcm5 wt (lane 1 vs 4). To know whether this deficiency is enough to compromise the recruitment of the MCM-Cdt1 complex, we repeated the experiment with the full complex. As shown in Fig. 1d, Mcm5 C-WHD is not required to obtain stable recruitment of the MCM-Cdt1 complex. However, when we repeated the same experiments with an Mcm7, which lacks the whole C-WHD (M7∆C1, Fig. 1e, f), we observed very different results. Although the differences are less significant with the trimer (Fig. 1e), with the full complex the recruitment of MCM-Cdt1 is drastically reduced if the Mcm7 C-WHD is not present (Fig. 1f). Interestingly, a shorter deletion that includes the last 41 amino acids, only present in *S. cerevisiae*, does not prevent the MCM-Cdt1 recruitment (Supplementary Fig. 2e).

Overall, these results show two findings: First, that the half hexamer composed by Mcm5/3/7 can be recruited in stoichiometric amounts by the OC. And second, that there is an essential requirement of Mcm7 C-WHD necessary for the recruitment of the MCM-Cdt1.

### Cdt1 is required to stabilize half MCM hexamer during recruitment

Different studies have shown that Cdt1 interacts with the half MCM hexamer (Mcm2/6/4) in solution^15,17^ and in the OCCM complex^8^. In addition, our previous data have shown that recruitment of MCM hexamer without Cdt1 is seriously compromised regarding this particular half MCM, while the recruitment of the other half, Mcm5/3/7, occurs normally^16^. To gain further insights into this differential recruitment between the two halves of the MCM, the ability of the OC to recruit a series of subcomplexes was tested, starting from the stoichiometric recruitment of the trimer Mcm5/3/7 up to the full MCM-Cdt1 heptamer.

**Fig. 2:**
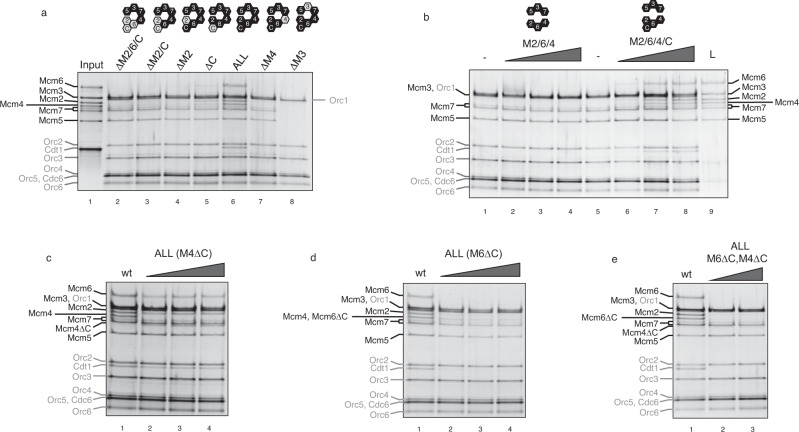
Cdt1 and C-WHDs of Mcm4 and 6 are required for half MCM recruitment. **a** Recruitment by the OC complex, starting with the tetramer Mcm5/3/7/4 (lane 2) up to the full heptameric MCM-Cdt1 complex (lane 6). The missing MCM-Cdt1 subunits, in lanes 2–5 and 7 and 8, are labelled with ∆ symbol and displayed as grey hexamers in the cartoon, while black hexamers indicate the subunits present in the assay. Negative controls of the MCM-Cdt1 recruitment are shown in lanes 7 (minus Mcm4) and 8 (minus Mcm3). 20% of the MCM-Cdt1 subunits used in the assay are shown in lane 1. **b** Two halves of MCM, Mcm5/3/7 and Mcm2/6/4, were isolated from gel filtration in separate runs and they were added separately and tested for recruitment. A constant amount of Mcm5/3/7 was used in combination with an increasing amount of Mcm2/6/4, without or with Cdt1 (lanes 2–4 and 6–8, respectively). The MCM-Cdt1 recruited in lane 8 was also tested for loading (ATP and high salt wash) (L, lane 9). **c** Recruitment of the MCM-Cdt1 either with Mcm4 wt (lane 1) or with increasing amounts of Mcm4 that lacks its C-WHD (M4∆C, lanes 2–4). **d** The same as **c** but with Mcm6 wt and Mcm6 lacking its C-WHD (M6∆C). **e** Recruitment of MCM-Cdt1 where both M6∆C and M4∆C are included. See also Supplementary Figs. 3 and 4.

It has been found that the progressive addition of Mcm4, 6, 2 and Cdt1 does not help to build up stoichiometric subcomplexes from the trimer Mcm5/3/7 (Fig. 2a, lanes 2–5, and Supplementary Fig. 3a), unless all four of them are included in the reaction (lane 6). In fact, Mcm4 alone recruits very poorly with Mcm5/3/7, as can be seen with the Mcm3-7 dimer, suggesting that the Mcm4-7 interface is somewhat unstable during recruitment. By contrast, it has been shown that both subunits can form a dimer in solution with significant ATPase activity^22^. It is likely that this is due to the conformational change of the half hexamer Mcm5/3/7 upon OC recruitment. In fact, the only combination that led to a residual recruitment of the half Mcm2/6/4 was that which did not include Cdt1 (lane 5). However, this residual sub-stoichiometric complex is not able to load MCM and is released upon ATP hydrolysis (Supplementary Fig. 3b). As important controls, first, the recruitment of all these subcomplexes is totally dependent on the presence of Mcm3 (Fig. 2a, lane 8), and second, the stabilization of the Mcm2/6/4/Cdt1 depends on Mcm4 (Fig. 2a, lane 7). Next, we asked whether both halves of the hexamer could be recruited separately. To investigate this, we purified both trimers via gel filtration and these were added separately in the preRC assay, with and without Cdt1 (Fig. 2b). In the absence of Cdt1, only the trimer Mcm5/3/7 was able to be recruited, regardless of the amount of the trimer Mcm2/6/4 used in the assay (lanes 2–4). However, the addition of Cdt1 allowed successful recruitment of MCM-Cdt1 by OC (lanes 6–8). Remarkably, this full complex, reconstituted by two halves, could still be loaded (lane 9).

Next, the role of the C-WHDs of Mcm4 and 6 was studied. Mcm4 C-WHD deletion affects to some extent MCM-Cdt1 recruitment. This said, a significant amount can still be seen to have been recruited, despite an underrepresentation of the tetramer Mcm2/6/4/Cdt1 (Fig. 2c). When we tested Mcm6 C-WHD (Fig. 2d), this tetramer was further underrepresented in the recruited complex. We envisaged two different ways in which these two WHDs could stabilize this tetramer: either by strengthening the interface between Mcm4 and 7 and/or contacting the OC, as shown in the OCCM structure^8^. Due to the fact that the co-deletion of both WHDs exacerbates the reduction in recruitment of this half MCM (Fig. 2e), together with the observation that the addition of these WHDs separately, does not improve the recruitment (Supplementary Fig. 4), we suggest that they contribute to the stabilization of the tetramer via their individual interactions with the OC.

Overall, these results show a dynamic MCM-Cdt1 heptamer that goes under major structural changes upon OC recruitment, mostly affecting the half MCM on to which Cdt1 binds, Mcm2/6/4. While half of the hexameric ring can be recruited by OC (Mcm5/3/7), regardless of the presence of the C-WHDs of Mcm4 and 6 or Cdt1, the recruitment of the other half requires the presence of Mcm6 C-WHD and Cdt1, and to a lesser extent Mcm4 C-WHD.

### Extended Cdt1 C-WHD is essential to Cdt1 preRC function

**Fig. 3:**
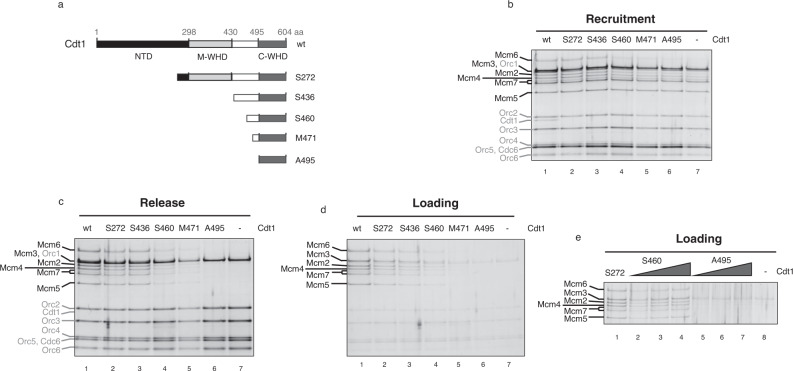
An extended Cdt1 C-WHD is required to recruit half MCM onto DNA. **a** Scheme illustrating the Cdt1 N terminus deletions used in the preRC assay. N terminus domain is shown in black (NTD), middle WHD in light grey (M-WHD) and C terminus WHD in dark grey (C-WHD). The long loop connecting both WHDs is shown in white (from amino acid 430–495). The first amino acid present in the Cdt1 truncations give the name to them. The S272 truncation contains a deletion of almost all NTD, while both domains, NTD and M-WHD, have been deleted in the S436 truncation. S460, M471 and A495 are different truncations affecting the length of the connecting loop and they are based on the OCCM structure (PDB: 5V8F). S460 truncation deletes mainly the loop section that interacts with Mcm2, while M471 keeps only the loop that interacts with Mcm6 C-WHD but not with the AAA+ domain. Finally, A495 deletes the extension of the helix 1 of Cdt1 C-WHD and abolishes any interaction with the Mcm6 C-WHD. **b** Recruitment of the MCM-Cdt1 by the OC complex (ATPγS and low salt washes), using Cdt1 wt (lane 1) or the different truncations (lanes 2–6). Negative control, where Cdt1 is omitted, is shown in lane 7. Under ATP hydrolysis, low salt wash is shown in **c** (release conditions) and high salt wash in **d** (loading conditions). **e** Loading of the MCM with increasing amounts of two different Cdt1 truncations (S460 and A495). The truncation that contains the smallest deletion (S272) is included as a positive control (Lane 1).

Cdt1 is composed of three domains: a non-conserved N-terminal domain and two conserved WHDs, middle (M) and C-terminal (C), connected by an extended and disordered loop (Fig. 3a). Recently, we solved the crystal structures of all these three domains^15^. To further investigate the role of these domains within preRC assembly, a series of Cdt1 truncations were made (Fig. 3a). We have previously shown that the N domain is important for origin licensing but not for MCM recruitment. We have used this fragment, starting with Serine 272, as the shorter truncation (S272), together with four larger deletions; (1) S436 removes the M-WHD but maintains the long linker between the M and C-WHD, (2) S460 deletion has a shorter linker, binding only to Mcm6, (3) M471 truncation contains only the linker section that binds Mcm6 C-WHD, and finally, (4) A495 construct lacks the extended part of helix 1 of the C-WHD. Thus, we removed all the interactions with the Mcm6 C-WHD present in the OCCM.

The effect of these specific Cdt1 deletions in the recruitment, release and loading of the MCM are presented in Fig. 3b–d, respectively. According to previous data, S272 has no effect on recruitment, despite a minor defect in loading. The deletion of the middle WHD of Cdt1 (S436) does not show increased changes on either recruitment or loading, compared to S272. However, the deletion of the extended loop between both WHDs of Cdt1 shows a decrease in both recruitment and loading, proportional to the removed section. This reduction is more evident under release conditions, because there is no residual binding of sub-stoichiometric complexes. This progressive reduction in loading stops with A495 (compare lanes 5 and 6 in Fig. 3d), which is unable to load MCM. In order to rule out any loading differences, due to amounts of proteins used, we tested the loading capacity of S460 and A495 with increasing amounts of them (Fig. 3e). While increasing amounts of S460 led to similar levels of MCM loading with S272, no loading was observed with A495, regardless of the amount used.

To summarize, out of the three domains of which Cdt1 is composed, only the C-WHD is essential in the stabilization of MCM recruitment by OC. Specifically, the minimum Cdt1 fragment able to recruit and load the wt MCM contains the extended C-WHD.

### Cdt1 stabilizes half MCM hexamer interacting with C-WHD of Mcm6

The interaction between Mcm6 C-WHD and Cdt1 C-WHD is well documented in both human and budding yeast^23–26^. Liu et al. described the key residues that interact between these two WHDs in human proteins using NMR. Due to the fact that most of these residues are conserved from yeast to humans, the authors could test their biological relevance in budding yeast. It was found that the mutated proteins were not able to load MCMs on chromatin and consequently the yeast cells could not progress through the S phase. In a different study, Fernandez-Cid et al. assessed the same set of mutations in the preRC context using purified proteins. The authors propose that Cdt1 allows an inhibitory role by Mcm6 C-WHD to be overcome and the mutations described by Liu et al. have a direct impact on this alleviation^14^. It is also worth mentioning that, in their assay, the initial recruitment showed significant dependence on Cdt1.

**Fig. 4:**
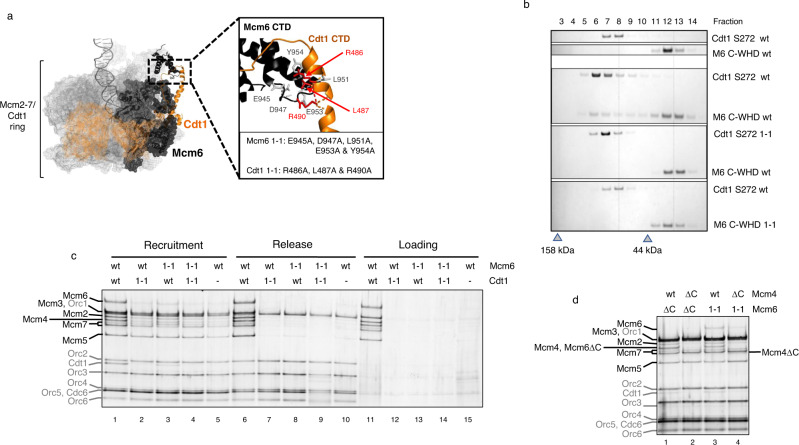
Stabilization by Cdt1 requires interaction with Mcm6 C-WHD. Characterization of the Cdt1 and Mcm6 subunits which contain mutations that affect their interaction via their C-WHDs. These mutations were originally described by Liu et al. and here are named 1-1 mutations. **a** MCM-Cdt1 structure in the recruited OCCM complex (PDB: 5V8F). Mcm2-7 subunits are represented in grey mesh, except Mcm6 that is represented in black, while Cdt1 is represented in orange. Only the secondary structure of the C-WHDs of Mcm6 and Cdt1 is illustrated (in black and orange, respectively). The zoomed area shows the residues substituted in the 1-1 mutations. **b** The interaction between Cdt1 and Mcm6, either wt or 1-1 mutations, is assessed by gel filtration. To see a bigger shift in the gel filtration profile, here we have used the Cdt1 S272 truncation (described in Fig. 3) and the Mcm6 C-WHD, either wt or 1-1 mutations. The peak fraction of two molecular weight markers is shown as a grey triangle (158 and 44 kDa). **c** PreRC assay of the MCM-Cdt1 complex, containing Mcm6 and Cdt1 wild type (wt) or 1-1 mutations (1-1). Omission of Cdt1 is used as negative control (lanes 5, 10 and 15). **d** Recruitment of MCM-Cdt1 containing an Mcm6 subunit with either 1-1 mutation (lanes 3 and 4) or a deletion of its C-WHD (∆C, lanes 1 and 2), in combination with Mcm4 wt or with its C-WHD deleted (∆C). Note that the Mcm4∆C and Mcm6∆C subunits are of reduced size and respectively migrate near Mcm7 and Mcm4 on SDS-PAGE.

Since we have not observed the same dependence, on either Cdt1 or Mcm6 C-WHD, with MCM-Cdt1 recruitment, we have tested the same mutations in the preRC assay to gain further insights (Fig. 4a). First, we tested the ability of these mutations to abolish their interaction by gel filtration. In order to see bigger shifts in the chromatographic profile, we only expressed the fragment S272 of Cdt1 (first 271 amino acids are deleted), either wt or with 1-1 mutations, together with both Mcm6 C-WHD wt and 1-1 mutations. As shown in Fig. 4b, neither the Cdt1 nor the Mcm6 containing this mutation could maintain the interaction with the corresponding wt partner. Our results confirm the findings of previously reported pull down assays, showing that these mutated subunits fail to physically interact^23,24^. When we tested them in the preRC assay, both showed a recruitment defect (Fig. 4c). Specifically, they were unable to recruit stoichiometric amounts of all six different subunits (compare lane 1 vs lanes 2–4). Again, we see an underrepresentation of the half hexamer (Mcm2/6/4) in both Cdt1 and Mcm6 1-1, being clearest in the Cdt1 1-1, as well as in combination with Mcm6 1-1, lanes 2 and 4, respectively. Importantly, none of these mutations showed the ability to load the MCM complex (lanes 12–14), In addition, the sub-stoichiometric complexes were released from origin DNA (lanes 7–9) under release conditions. Therefore, the residual MCM-Cdt1 complex observed, especially in Mcm6 1-1 (lane 3), probably does not contain the DNA inside its channel and nor does it reflect a partial defective mutation. Both the loading and release show clearly that 1-1 mutations do not form DHs. We suggest that these residual complexes are probably bound to OC by the C-WHD of Mcm4 and 6 without duplex DNA inside the MCM. Accordingly, when we combined the Mcm4 C-WHD deletion with Mcm6 1-1, the Mcm2/6/4/Cdt1 tetramer was reduced to similar levels to that of the double deletion of the C-WHDs of Mcm6 and Mcm4 subunits (Fig. 4d, compare lanes 2 and 4).

To summarize therefore, these experiments show that point mutations, which abolish the interaction between the C-WHDs of Cdt1 and Mcm6, have the same hallmarks that have already been shown when neither Cdt1 nor Mcm6 C-WHD are included. These are specifically: (1) recruitment of a sub-stoichiometric MCM complex, where half hexamer (Mcm2/6/4) is underrepresented, (2) release from DNA by the QC, and (3) inability to load MCM.

### Orc5 interaction by Mcm6 C-WHD latches the MCM-Cdt1 with the OC

**Fig. 5:**
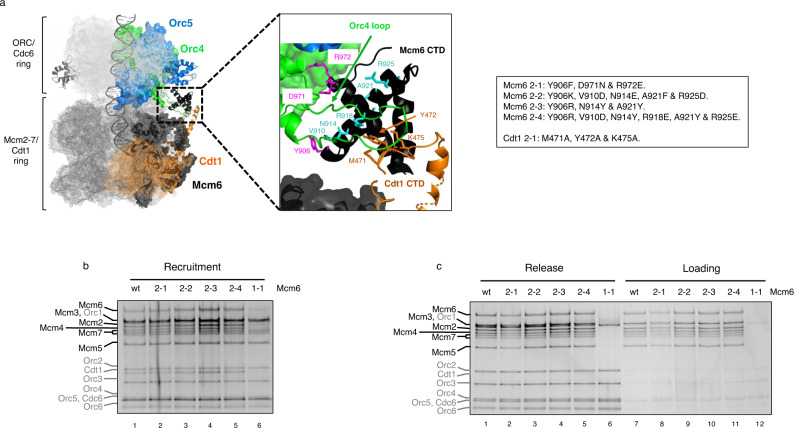
Interaction with the Orc4 loop by Mcm6 C-WHD is not essential for Cdt1 stabilization of half MCM. **a** Cryo-EM structure of the OCCM complex^8^. The ORC-Cdc6 ring is illustrated in light grey mesh, while the MCM-Cdt1 ring is in dark grey mesh. The surface of specific individual proteins are highlighted in different colours: Orc 4 (green), Orc5 (blue), Mcm6 (black), and Cdt1 (orange). Of these, the domains studied in this work are further highlighted with their secondary structure, maintaining the colours (extended C-WHD of Cdt1, C-WHD of Mcm6, Orc4 C-WHD and Orc5 AAA lid). Note that the secondary structure of Mcm3 C-WHD is also illustrated in grey. The zoomed area shows in more detail the interaction between the Orc4 loop (shown in green) and the C-WHDs of Mcm6 and Cdt1 (shown in black and orange, respectively). Three different amino acid substitutions, in the C-WHDs of Mcm6 and Cdt1, affecting the interaction with the Orc4 loop were studied. First, contacts at the beginning and the end of the Orc4 loop by the C-WHD of Mcm6 were mutated (Mcm6 2-1, shown in magenta in the zoomed section). Second, different residues present in the main helix of Mcm6 that establish contacts with Orc4 loop were substituted with different amino acids going from mild to severe changes (Mcm6 2-2, 2-3 and 2-4, shown in cyan. See also Supplementary Fig. 5). Third, the contacts between the Orc4 loop and the C-WHD of Cdt1 were mutated in Cdt1 2-1 (in orange). Specific substitutions present in the different proteins mutated are summarized in the box. **b** Recruitment of MCM-Cdt1, containing Mcm6 wt, 2-1, 2-2, 2-3 and 2-4 mutations (lanes 1-5). Mcm6 1-1 is included as negative control in lane 6. **c** Release and loading of the complexes analysed in **b**. The recruitment and loading of the MCM-Cdt1 complex containing Cdt1 2-1 mutation is shown in Supplementary Fig. 6a, b.

**Fig. 6:**
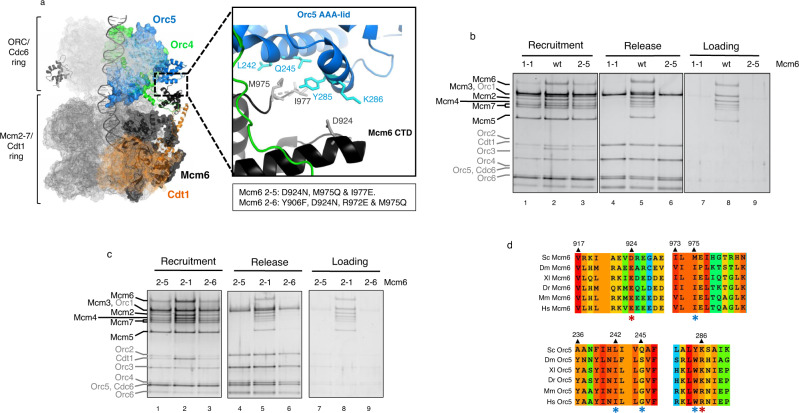
Interaction with the AAA-lid domain of Orc5 by Mcm6 C-WHD is essential for Cdt1 stabilization of half MCM. **a** Cryo-EM structure of the OCCM complex^8^. The ORC-Cdc6 ring is illustrated in light grey mesh, while the MCM-Cdt1 ring is in dark grey mesh. The surface of specific individual proteins are highlighted in different colours: Orc 4 (green), Orc5 (blue), Mcm6 (black), and Cdt1 (orange). Of these, the domains studied in this work are further highlighted with their secondary structure, maintaining the colours (extended C-WHD of Cdt1, C-WHD of Mcm6, Orc4 C-WHD and Orc5 AAA lid). Note that the secondary structure of Mcm3 C-WHD is also illustrated in grey. The zoomed area shows in detail the interaction between the Mcm6 C-WHD (in black) and the Orc5 AAA+lid domain (in blue). Side chains of residues studied are depicted in grey (Mcm6) and cyan (Orc5). Specifically, D924 of Mcm6 forms an ionic pair with K286 of Orc5, while M975 of Mcm6 interacts with a hydrophobic patch of Orc5, composed by L242, Q245 and Y285 (Supplementary Fig. 7). In addition, the position of a semiconserved I977 in Mcm6 is also illustrated. The specific amino acid substitutions in Mcm6, containing 2-5 and 2-6 mutations, are detailed in the box. Note that 2-6 mutations include two different mutations already present in 2-1 (Y906 and R972) and in 2-5 mutations (D924 and M975). **b** PreRC assay with MCM-Cdt1 containing Mcm6 wt, 1-1 and 2-5 mutations. **c** PreRC assay comparing Mcm6 2-5 and 2-6 mutations. Additionally, Mcm6 2-1 is also included because some mutations present in Mcm6 2-6 are also present in Mcm6 2-1. **d** Alignment of the residues involved in the interaction between the Mcm6 C-WHD and the Orc5 AAA+-lid. The ionic pairs between Mcm6 D924 and Orc5 K286 are highlighted in red asterisks. The structural conservation between the hydrophobic patch of Orc5 (L242, Q245 and Y285) and Mcm6 M975 is highlighted in blue asterisks. Sc *Saccharomyces cerevisiae*, Dm *Drosophila melanogaster*, Xl *Xenopus laevis*, Dr *Danio rerio*, Mm *Mus musculus*, Hs *Homo sapiens*.

In the OCCM complex, the Mcm6 C-WHD domain assumes an extended conformation. Structural studies have shown that, while in solution, this domain sits on top of the MCM ring, away from the Cdt1 C-WHD, in the OCCM structure, Mcm6 C-WHD moves towards Cdt1 C-WHD, which expands significantly the length of the helix 1 of the WHD. This in turn allows further interactions between both domains^8,21^. In the OCCM structure^8^, Mcm6 C-WHD interacts with the OC, just on the opposite side where Mcm3 C-WHD interacts, through Orc4 and 5 interactions (Fig. 5a). *S. cerevisiae* ORC4 C-WHD have two specific insertions, not present in metazoans. (1) An alpha helix that has been shown to make critical contacts with origin DNA^27^ and (2) a long loop that interacts extensively with Mcm6 C-WHD, and to a lesser extent with the Cdt1 C-WHD, as shown in the zoomed area of Fig. 5a. Regarding the Orc5 interaction, Mcm6 is contacting the AAA-lid domain, which sits just above the Orc4 WHD in the OCCM structure (zoomed area in Fig. 6a). Both of these contacts could explain why an extended version of C-WHD of Cdt1 is required to stabilize half of the helicase. To further investigate their contribution to this stabilization, we expressed a series of Mcm6 C-WHD mutations affecting either Orc4 loop (Fig. 5) or Orc5 AAA-lid interactions (Fig. 6).

To abolish the interactions with the Orc4 loop, we designed three types of mutations (described in Fig. 5a box). (1) In the Mcm6 2-1 substitution, we substituted the amino acids that make contact at the beginning and the end of the Orc4 loop (shown in magenta). (2) Three more mutations contain multiple amino acid substitutions in the alpha helix, which make extensive contacts with the loop itself (shown in cyan). These range from mild to severe changes, based on the physicochemical properties of the substituted residues (Supplementary Fig. 5). Specifically, in Mcm6 2-2, 5 out of these 6 residues were mutated with rather semiconservative amino acid-type changes. By contrast, in Mcm6 2-3 and 2-4, we changed three and six residues, respectively, but with very different side chains properties. (3) Three amino acids from Cdt1 that interact with the apex region of Orc4 loop were also mutated (shown in orange). Surprisingly, none of these substitutions exhibited a defect in MCM recruitment (Fig. 5b and Supplementary Fig. 6a). In fact, they appeared able to load the helicase as well as the wt proteins (Fig. 5c and Supplementary Fig. 6b). In this assay, we have used the Mcm6 1-1 mutation, as a negative control. A reproducible mild reduction in loading can be noticed when using Mcm6 2-1 substitution (compare lanes 7 and 8 of Fig. 5c). We suggest that this small decrease is due to a slight reduction in protein’s solubility, due to the observation of slightly lower expression levels than wt. Nevertheless, the differences with the Mcm6 1-1 are striking.

Mcm6 C-WHD also makes contacts with the AAA-lid domain of Orc5 (Fig. 6a zoomed area and Supplementary Fig. 7). While Mcm6 D924 and Orc K286 make an ionic pair interaction, the M975 contacts a hydrophobic patch of Orc5 AAA+lid domain (L242, Q245 and Y285). In addition to these two residues, a semiconserved I977 was also mutated in Mcm6 2-5. Remarkably, this mutated subunit showed deficiency in recruitment, similar to the Mcm6 1-1 mutation. Here the same half hexamer was recruited at similar levels to the wt proteins (Mcm5/3/7), while the other half was reduced (Mcm2/6/4/Cdt1). In agreement with the results that we have shown previously, this sub-stoichiometric complex was seen to release from origin DNA and showed no ability to load the MCM under ATP hydrolysis (Fig. 6b). The fact that Mcm6 2-5 had recruited more Mcm2/6/4/Cdt1 than Mcm6 1-1 does not mean that this mutation is partially defective, because the loading defect is very clear and it releases completely from DNA under ATP hydrolysis (Fig. 6b, lane 6). As happens with Mcm6 1-1, Mcm6 2-5 in combination with the deletion of the C-WHD of Mcm4, reduces the residual binding of the tetramer Mcm2/6/4/C significantly (Supplementary Fig. 6c). Therefore, it is unlikely to contain duplex DNA within.

To further narrow down the key residues that contact Orc5, a sixth mutation in the Mcm6 subunit was made (Mcm6 2-6). In this mutation, we combined two residues mutated in Mcm6 2-1 (Y906 and R972) and two mutated residues in Mcm6 2-5 (D924 and M975). Since the Mcm6 2-1 mutation is able to recruit and load as the wt, together with the fact that Mcm6 2-6 is as deficient as Mcm6 2-5 in the preRC assembly (Fig. 6c), both observations lead us to exclude the residue I977 as playing an important role in this interaction. This, therefore, narrows down the key residues involved in the interaction between Mcm6 C-WHD and Orc5 AAA+lid to D924 and M975 of Mcm6 (Supplementary Fig. 7). Just as has been noticed with the critical residues contacting C-WHDs of Cdt1 and Mcm6, the ionic pair interaction between Mcm6 D924 and Orc5 K286 is conserved through evolution (Fig. 6d, red asterisks in Mcm6 and Orc5), as it is the structure of the hydrophobic patch in Orc5 (L242, Q245 and Y285) where Mcm6 M975 binds (Fig. 6d, blue asterisks in both Mcm6 and Orc5).

## Discussion

**Fig. 7:**
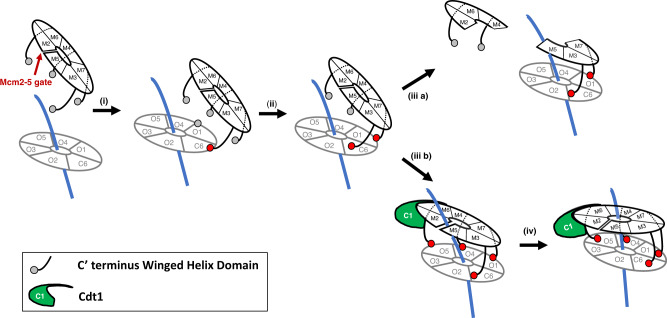
Model proposed for MCM-Cdt1 recruitment by the OC complex. (i) ORC-Cdc6 bound to duplex DNA is represented as a grey ring (OC complex). Orc subunits are labelled with an O+ subunit number and Cdc6 as C6. Orc6 is omitted because it does not participate in the ring conformation. Double-strand DNA is shown in blue. The MCM complex is illustrated as a black ring. Individual subunits are labelled with an M+ subunit number. Neighbouring subunits Mcm2 and 5 are not in contact, illustrating the Mcm2-5 gate (red arrow). In addition, C-WHDs of Mcm3, 7, 4 and 6 are shown as grey circles if they do not contact the OC or in red, if they do contact. Note that Mcm2 does not contain a WHD in *S. cerevisiae* and Mcm5 C-WHD is omitted for clarification purposes, since we have shown that, if deleted in the MCM-Cdt1 complex, it recruits as effectively as the wt (Fig. 1d). (ii) The first contact between the OC and the MCM-Cdt1 complexes is mediated by the Mcm3 C-WHD (red circle). (ii) The first contact is followed by the Mcm7 C-WHD, establishing directionality to the whole interaction. (iii a) If an extended Cdt1 C-WHD (in green, labelled C1) is not present, or has mutations affecting the interaction with Mcm6 C-WHD (Cdt1 1-1), or Mcm6 C-WHD contains mutations affecting either Cdt1 C-WHD (Mcm6 1-1) or Orc5 AAA-lid (Mcm6 2-5 and 2-6), the insertion of dsDNA through the Mcm2-5 gate destabilizes half MCM hexamer and only the trimer Mcm5/3/7 can be recruited by the OC complex. However, if an extended C-WHD of Cdt1 is present, the full hexamer can be stabilized upon duplex DNA entry (iii b). Finally, both rings can be stacked onto one another with dsDNA running through them, as seen in the OCCM structure, with the collaboration of the C-WHDs of Mcm4 and 6 (iv).

MCM constitutes the core of the replicative helicase. How the loading machinery (ORC-Cdc6 complex and Cdt1) manages to insert dsDNA inside the MCM channel is an important question that remains to be answered. The data presented here support the following model (Fig. 7).

In agreement with previous data^11,16^, the first contact between the MCM-Cdt1 complex and the OC complex is mediated by Mcm3 C-WHD (step i). Here we have shown that, if Mcm3 is not include in the recruitment, neither the individual subunits nor different subcomplexes can be recruited by the OC.

The first landing of Mcm3 C-WHD is followed by the C-WHD of Mcm7 (step ii). The binding of this second WHD gives the right directionality towards the stacking of both rings, MCM-Cdt1 and ORC-Cdc6. In fact, recent cryo-EM studies have discovered and characterized some intermediates before the OCCM complex^10^. Miller et al. have shown a complex named OC-MC, where MCM-Cdt1 is contacting OC but is not completely engaged. Importantly, dsDNA is poised for threading into the MCM channel, because it is aligned with the Mcm2-5 gate and dsDNA is bent by ORC. Similar results were obtained with the semi-attached complex described recently by Yuan et al.^28^. These authors also studied the localization of Mcm3 and 7 C-WHD. Perhaps unsurprisingly, they show their interaction to be nearby the Cdc6. This would explain that, without Cdc6, ORC is unable to recruit the MCM-Cdt1. Despite the essential contribution of both Mcm3 and 7, we repeatedly observed that the most stable subcomplex recruited onto DNA was the half hexamer composed of Mcm5/3/7. Because we have not seen recruitment changes with Mcm5 minus C-WHD, this further stabilization of the trimer recruitment could be caused by the binding of Mcm5 to Mcm3.

Once the OC and the MCM-Cdt1 rings are latched by Mcm3 and 7, to finish their coupling and lead to the OCCM formation, a major problem has to be overcome. That of the DNA interference. Indeed, here we show that the DNA insertion into the MCM channel induces conformational changes that destabilize the weaker interface after the Mcm2/5 gate, the Mcm7/4. This interface sits opposite to the Mcm2/5 gate, and if Cdt1 is not present during recruitment (iii a), its destabilization splits the MCM into two halves.

These structural changes associated with the DNA intake, either by the DNA or the C-WHDs of the different Mcm subunits, induce the expansion of the helix 1 of Cdt1 C-WHD, required to interact with the Mcm6 C-WHD. Once this interaction takes place, Mcm6 C-WHD is able to contact the Orc5 AAA+lid domain and latch between the Cdt1 and the OC. In conclusion, these multiple interactions allow the stabilization of the MCM upon DNA entry by Cdt1 (iii b). In agreement with this model, when the key conserved residues of the interactions were mutated between C-WHDs of Cdt1-Mcm6, or Mcm6-Orc5 AAA-lid, only the first half hexamer that contacts the OC was recruited stoichiometrically (Mcm5/3/7). Interestingly, all the key residues present in Cdt1, Mcm6 and Orc5 involved in this stabilization are conserved in the metazoans. Their conservation suggests a general mechanism on how eukaryotic cells incorporate dsDNA into the core of their replicative helicase. Finally, we cannot rule out completely that the Orc4 loop plays also a role in this stabilization, because although we have introduced important mutations to abolish its interaction with Mcm6 C-WHD, the length and the flexibility of the loop might still allow a structural re-arrangement that might preserve the interaction with Mcm6 C-WHD.

Previous structural studies have shown that, during the OCCM assembly, the MCM-Cdt1 complex goes from a spiral to a planar conformation^15^. Our proposition therefore is that the different Mcm’s C-WHDs, starting with Mcm3 and followed by Mcm7, 4 and 6, respectively, act as a zipper during the coupling of the OC and the MCM-Cdt1 complexes that ultimately results in the OCCM formation (iv).

In this study, we have used multiple MCM-Cdt1 mutations that challenge the preRC assay in different ways: point mutations, deleted subunits, and multiple subcomplexes. Interestingly, all mutated proteins were released from origin DNA under release or QC conditions. These results significantly expand the range, where an ATPase-dependent QC ensures the maintenance of functional replication origins along S phase.

Two examples where DNA incorporation has been studied extensively are DnaB helicase and clamp loader. In both cases, the loader stabilizes an open form in solution that allows DNA entry. However, the MCM loading departs from this canonical clamp loading, because most of the loaders (ORC and Cdc6) are already bound to DNA^29^. An important consequence of this is that MCM-Cdt1 ring has to overcome the interference by DNA during its coupling to the OC. Here we present a model where this topological problem during DNA insertion is overcome by the only helicase loader not bound to DNA, Cdt1.

## Methods

All oligonucleotides are described in Supplementary Table 1, all plasmids in Supplementary Table 2 and a comparison between wt and mutated proteins is given in Supplementary Table 3.

### PreRC assay with purified proteins

#### Proteins

ORC and Cdc6 proteins were purified as described previously^16^.

Most of the MCM-Cdt1 proteins (including mutated proteins) used in this work were obtained without extra amino acids, as native proteins. Using the Champion^TM^ pET SUMO Protein Expression System (Invitrogen), a small ubiquitin-like modifier (SUMO) was fused to the N-terminal of the protein. The cleavage by SUMO protease results in the production of native protein, without extra amino acids. Expression and purification protocols are included as Supplementary Data.

Once the different subunits of the MCM-Cdt1 complex were purified individually, we tested them in two different ways: either by mixing them and purifying the MCM-Cdt1 complex via gel filtration before their addition into the preRC assay or by adding individual subunits straight into the assay. Both sources of MCM-Cdt1 show similar results in recruitment and loading, as shown in Supplementary Fig. 2b. Despite slight differences in the amounts of different subunits when added independently (Supplementary Fig. 2a), the recruited and loaded complexes maintain the stoichiometry between the different subunits. These results illustrate the robustness of the preRC assay with purified proteins. Both sources of MCM have been used interchangeably in this work.

#### DNA template

Linear DNA containing ARS305 were generated by PCR using ARS305-F-PC-Bio-Eco and ARS305-R primers, using a plasmid containing ARS305 as a template. The 5’ primer contains a photocleavable biotin (IDT). PCR products were purified using the Monarch PCR&DNA Cleanup Kit (NEB). Three hundred nanograms of purified PCR were coupled to 3 μl slurry Dynabeads M-280 streptavidin magnetic beads (Life Technologies).

#### PreRC assembly assay

All reactions were prepared on ice in a final volume of 40 μl of loading buffer (25 mM HEPES-KOH pH = 7.6, 10 mM MgOAc, 0.02% IGEPAL CA630 (Sigma), 5% Glycerol and 100 mM KOAc). Reactions contained either 5 mM ATP (Sigma) or ATPγS (Sigma) as noted. All reactions contained 2 μl of DNA beads. Fifty nanomolar ORC and 50 nM Cdc6 were added in this order to allow the formation of the OC complex. Individual MCM-Cdt1 subunits or different complexes were added at 250 nM after ORC and Cdc6 addition. Reactions were immediately transferred to 30 °C with mixing at 1100 RPM for 20 min. Reactions were then washed either twice with a low salt wash (25 mM HEPES-KOH pH = 7.6, 5 mM MgOAc, 1 mM EDTA, 1 mM EGTA, 0.02% IGEPAL CA630, 10% Glycerol and 300 mM KOAc) or just once with low salt wash followed by high salt wash (same as low salt wash but with 500 mM NaCl instead of 300 mM KOAc). Samples were then resuspended in 10 μl low salt wash buffer and photocleaved by exposure to ultraviolet (310 nm) for 2.5 min on ice. DNA-bound proteins were mixed with sample buffer and analysed by sodium dodecyl sulfate–polyacrylamide gel electrophoresis.

The preRC assembly with purified proteins has three major outcomes: recruitment, release or QC, and loading (Supplementary Fig. 1a), depending on the ATP source and the wash use after preRC assembly (described in Table 1 of Supplementary Fig. 1). Recruitment conditions implies the use of slow hydrolysable ATP analogue (ATPγS) and low salt washes. Under these conditions, all preRC members are bound to origin DNA, assembling a complex known as OCCM. However, if we allow ATP hydrolysis, using ATP instead of ATPγS, then we can differentiate between loading and release, depending on the salt wash used. With high salt wash, only the double hexamer is stable enough to remain bound to the origin DNA (loading conditions). On the other hand, with low salt washes, ORC is also retained with the origin DNA, together with the double hexamer (release conditions). In addition, under these release conditions, if the criteria for double hexamer formation is not met, an ATPase-dependent QC allows the irreversible release of the individual MCM-Cdt1 subunits or subcomplexes, and only ORC remains bound to the DNA.

### Statistics and reproducibility

All the experiments were repeated at least three times.

### Reporting summary

Further information on research design is available in the Nature Research Reporting Summary linked to this article.

## Supplementary information

Supplementary Information

Reporting Summary

## Data Availability

All relevant data are available from the authors upon reasonable request or can be found in the Source data file. Source data are provided with this paper.

## Source data

Source Data
