# Supplementary material for: Stabilisation of half MCM ring by Cdt1 during DNA insertion
Source: Nat Commun. 2021 Mar 19;12:1746. doi: 10.1038/s41467-021-21932-8 (PMC7979726; doi:10.1038/s41467-021-21932-8)
Supplement: Supplementary file 1 — Supplementary Information [file 41467_2021_21932_MOESM1_ESM.pdf]

Vectors

Mcm subunits.

Mcm2 was amplified from *S. Cerevisiae* genomic DNA, using MG1-2 primer pairs, and cloned into Champion™ pET SUMO (Invitrogen) (pMG1).

Mcm3 was amplified from *S. Cerevisiae* genomic DNA, using MG3-4 primer pairs, and cloned into Champion™ pET SUMO (pMG2).

Mcm4, *Mcm4ΔC,* and *Mcm4 C-WHD* were amplified from *S. Cerevisiae* genomic DNA using MG5-6, 5-19, and 20-6 primer pairs. These PCR products were cloned into Champion™ pET SUMO generating pMG3, pMG7, and pMG8, respectively.

Mcm5 and *Mcm5ΔC* were amplified from *S. Cerevisiae* genomic DNA using MG7-8 and 7-21 primer pairs. These PCR products were cloned into Champion™ pET SUMO leading to pMG4 and pMG9, respectively.

Mcm6, *Mcm6 C-WHD*, and *Mcm6ΔC* were amplified from *S. Cerevisiae* genomic DNA using the following primer pairs: MG9-11, 23-12, and 10-22. *Mcm6* and *Mcm6 C-WHD* were cloned into pet28hisSUMO using the BamHI site (pMG5), and the BamHI and XhoI sites (pMG11), respectively. *Mcm6ΔC* was cloned into pCP6HP using the XmaI and BamHI sites (pMG10). *Mcm6 C-WHD 1-1* was amplified from pMG21 as a template DNA, using MG23-12 primer pairs. This PCR product was cloned into pet28hisSUMO, between BamHI and XhoI sites (pMG12). *Mcm6 1-1* (pMG21) was synthesized by Thermofisher and cloned into pet28hisSUMO between AarI and KpnI sites. *Mcm6 2-1* (pMG22), *Mcm 6 2-2* (pMG23), *Mcm6 2-3* (pMG24), *Mcm6 2-4* (pMG25), *Mcm6 2-5* (pMG26), and *Mcm6 2-6* (pMG27) were also synthesized by Thermofisher, and cloned into pet28hisSUMO between the SwaI and KpnI sites.

Mcm7ΔC1 and *Mcm7ΔC2* were amplified from *S. Cerevisiae* genomic DNA using MG13-24 and 13-25 primer pairs. These PCR products were cloned into Champion™ pET SUMO leading to pMG13 and pMG14, respectively.

Cdt1.

Cdt1 with an additional 3xFlag, *Cdt1 S436, Cdt1 S460*, and *Cdt1 M471* were amplified from *S.Cerevisiae* genomic DNA, using MG15-16, 18-27, 28-17, and 29-17 primer pairs. *Cdt1 Flag* was cloned between BamHI and NotI sites of pet28hisSUMO (pMG6), while *Cdt1 S460* and *Cdt1 M471* were cloned between BamHI and XhoI sites, yielding to pMG15 and pMG16 respectively. Cdt1 S436 was cloned into Champion™ pET SUMO (pMG18). *Cdt1 S272 1-1* was amplified from pMG17 as a template DNA, using MG26-16 primer pairs, and cloned into pet28hisSUMO, between BamHI and NotI sites (pMG19). *Cdt1 1-1* (pMG17) and *Cdt1 2-1* (pMG20) were synthesized by Thermofisher and cloned into pet28hisSUMO. *Cdt1 1-1* was inserted between BsrgI and NotI sites, while *Cdt1 2-1* was inserted using SacI and NotI restriction sites.

Expression and purification

Buffers.

Buffer D contains 0.7M NaCl and 25mM Tris-HCl pH 7.6. Buffer E is composed by 25mM Tris-HCl pH 7.6. Buffer F 25mM Tris-HCl pH7.6 and 10% glycerol. Buffer G contains 0.3M Kacetate, 25mM HEPES pH 7.6 and 10% glycerol.

Purification of ORC from ySD-ORC.

We followed the protocol described by Frigola et al., 2013. However, with the last two chromatographic columns were swapped. First, we concentrated the protein with the MonoQ^{TM} 5/50 GL (GE Healthcare) and second, we changed the buffer with the Superdex200 Increase 10/300 GL (GE Healthcare) pre-equilibrated in buffer C/0.3M Kacetate (buffer stated at the original protocol).

Proteins expressed in bacteria.

All expression plasmids were transformed into BL21 DE3 Codon+RIL cells (Agilent). 2.8L of cells (unless indicated) were grown with LB at 37ºC to a density of OD_{600}=0.5-0.8. Cells were cooled down, and then IPTG was added to 1mM. Induction was carried out 7h at 20ºC (unless indicated). Cells were harvested by centrifugation for 5min at 4500rpm using a Thermo Scientific 75003180 rotor, and stored at -80ºC.

- *GST-Cdc6.*

We followed the protocol described by Frigola et al., 2013 with minor changes. The preScission protease (GE Healthcare) was incubated overnight instead of 2h at 4ºC. Also, the peak fractions resulting from the hydroxyapatite elution were not concentrated using a centricon before being aliquoted.

- *HIS-Mcm2.*

The pellet was resuspended in 80ml of buffer D+1mM PMSF, and 1mM lysozyme (hereafter, lysis buffer). Cells were kept on ice and disrupted using sonication (Labsonic). The sample was centrifuged at 10000rpm for 30min at 4ºC with a Thermo Scientific Fiberlite F13-14x50cy rotor. The soluble phase containing the his_6-tagged Mcm2 was incubated on a 4ml His Trap FF beads (Generon) at 4ºC for 30min. Beads were washed with 20 column volumes (CVs) of buffer D+10mM imidazole. The protein was eluted with buffer D+200mM imidazole. His_6-SUMO tag from Mcm2 was removed by digestion with 500ng of Ulp1 protease on ice for 15min. 5ml of untagged protein was subjected to fractionation over a HiLoad^{TM} 16/600 SuperdexTM 200pg (GE Healthcare) pre-equilibrated in buffer D. Peak fractions were pooled, diluted 1:4 with buffer E, and concentrated over a 1ml MonoQ^{TM} 5/50 GL column, using an elution gradient of 0.17-0.7M NaCl in buffer F, over 1CV. Peak fractions containing Mcm2 were aliquoted, snap-frozen in liquid nitrogen, and stored at -80ºC.

- *HIS-Mcm3.*

Basically, like HIS-Mcm2 with minor changes. The lysis buffer also contained 2 tablets of protease inhibitor cocktail (Roche). Moreover, the protein was diluted 1:5 with buffer E before being applied

to the MonoQ^{TM} 5/50 GL column, and the elution was performed with a step from 0.14 to 0.7M NaCl in buffer F.

- *HIS-Mcm4.*

Essentially like HIS-Mcm2 with some changes after the HiLoad^{TM} 16/600 Superdex^{TM} 200 pg column. Fractions containing HIS-Mcm4 were injected to a second HiLoad^{TM} 16/600 Superdex^{TM} 200 pg column pre-equilibrated in buffer G. Peak fractions were pooled, concentrated with a 30K MWCO centricon (Generon), aliquoted, snap-frozen in liquid nitrogen, and stored at -80ºC.

- *HIS-Mcm4ΔC.*

The starting cultures were 1.5L in volume. To purify this protein, a similar protocol to the purification of HIS-Mcm2 was followed, except that before applying the protein to the MonoQ^{TM} 5/50 GL column a dilution of 1:7 with buffer E was made. Also, the column was eluted with a liner 0.1-0.5M NaCl gradient in buffer F over 5CVs.

- *HIS-Mcm4 C-WHD.*

The starting cultures were 0.75L in volume. The purification was performed as described for HIS-Mcm2 until the Ulp1 digestion, because the protein is smaller, and therefore, HIS tag and Ulp1 cannot be separated from the protein of interest by gel filtration. So, after the protease cleavage, a 1:10 dilution was made with buffer D to dilute the imidazole. Then, the protein was loaded onto a 5ml HisTrap^{TM} FF crude (GE Healthcare) column pre-equilibrated in buffer D+10%glycerol. The flow-through containing the Mcm4 C-WHD was collected, snap-frozen in liquid nitrogen, and stored at -80ºC. To elute the retained HIS tag and the Ulp1 protease of the 5ml HisTrap^{TM} FF crude column, buffer D+200mM imidazole was injected into the column.

- *HIS-Mcm5.*

Fundamentally like HIS-Mcm2 with several changes. All the buffers used for this protein also contained 0.05% IGEPAL (substitute of NPGO). The pellet was resuspended with a lysis buffer containing 1M NaCl instead of 0.7M NaCl, and an additional 1mM DTT. Furthermore, after the His Trap FF elution, 1mM EDTA, 1mM EGTA, 1mM PMSF, and 1mM DTT was added to the protein. Plus, in this case, the MonoQ^{TM} 5/50 GL elution was performed with a 0.15-0.7M NaCl gradient in buffer F over 8CVs.

- *HIS-Mcm5ΔC.*

The starting cultures were 1.5L in volume. Like HIS-Mcm5 with some changes at the end of the process. After the Ulp1 digestion, the protein was fractionated onto a Superdex^{TM} 200 Increase 10/300 GL pre-equilibrated in buffer D+10% glycerol, and no MonoQ column was performed. Peak fractions were aliquoted, snap-frozen in liquid nitrogen, and stored at -80ºC.

- *HIS-Mcm6.*

Essentially like HIS-Mcm2 with some changes. All the buffers used for this protein also had 1mM ATP, 5mM $Mg(OAc)_2$, and 1mM EDTA. The lysis buffer also contained 2 tablets of protease inhibitor cocktail. Additionally, after the His Trap FF beads elution, 1mM DTT, 1mM PMSF, and 1mM EGTA were added to the protein. Plus, before applying the protein to the MonoQ^{TM} 5/50 GL

column a dilution of 1:2.7 with buffer E was made. The column was eluted with a step from 0.25 to 0.7M NaCl in buffer F.

- *HIS-Mcm6ΔC.*

The starting cultures were 1.5L in volume. Exactly like HIS-Mcm6 but the digestion was made with 3C protease instead of Ulp1.

- *HIS-Mcm6 C-WHD and HIS-Mcm6 C-WHD 1-1.*

A similar protocol to the purification of HIS-Mcm4 C-WHD was followed, except that the induction was carried out 5h at 30ºC.

- *HIS-Mcm6 1-1, HIS-Mcm6 2-1, HIS-Mcm6 2-2, HIS-Mcm6 2-3, HIS-Mcm6 2-4, HIS-Mcm6 2-5, and HIS-Mcm6 2-6.*

The starting cultures were 1.5L in volume. The protocol was the same as the one described for HIS-Mcm6.

- *Mcm7.*

We generally followed the protocol described by Davey et al., 2003 but with some differences. The buffers used are the same as the original protocol. The starting cultures were 2.8L in volume. For cell lysis, the pellet was resuspended with 45ml with a final concentration of 1MNaCl, 2mM DTT, 1mM PMSF, 1mM lysozyme, 50mM Tris-HCl pH 7.6. Cells were lysed by sonication and centrifuged like HIS-Mcm2. The supernatant was treated with 0.3mg/ml ammonium sulfate. After centrifugation for 30 min at 10000rpm at 4ºC, the pellet was resuspended in buffer A containing 0.25mg/ml ammonium sulfate and then centrifuged as above. This step was repeated using buffer A containing 0.2mg/ml ammonium sulfate. The resulting pellet was resuspended in 12ml of buffer A and dialyzed against buffer A for 1h. The protein was loaded onto a 5ml HisTrap^{TM} Q FF column (GE Healthcare) pre-equilibrated in buffer A+70mM NaCl. The column was eluted with 50ml, 70mM-500mM NaCl gradient in buffer A. Peak fractions containing the protein were dialyzed overnight against buffer B+90mM NaCl. Mcm7 was applied to a 2ml HiTrap^{TM} Heparin HP column (GE Healthcare) pre-equilibrated in buffer B+90mM NaCl. The column was eluted with a 20ml, 90mM-500mM NaCl gradient in buffer B. Peak fractions containing the protein were pooled. A 1:3 dilution was made with buffer A. Then, the sample was injected into a 1ml MonoQ^{TM} 5/50 GL column pre-equilibrated in buffer A+100mM NaCl. Bound proteins were eluted with a 3ml, 100mM-1M NaCl gradient in buffer A. Peak fractions were then injected to a 24ml Superdex^{TM} 200 Increase 10/300 pre-equilibrated in 0.3M Kacetate, 25mM HEPES pH 7.6, and 10% glycerol buffer. Peak fractions containing Mcm7 were pooled, aliquoted, snap-frozen in liquid nitrogen, and stored at -80ºC.

- *HIS-Mcm7ΔC1.*

The starting cultures were 1.5L in volume. Essentially like HIS-Mcm2 with several changes at the end of the process. After the Ulp1 protease digestion, the protein was fractionated on a Superdex^{TM} 200 Increase 10/300 pre-equilibrated in buffer F, and no MonoQ column was performed. Then, peak fractions were pooled, aliquoted, snap-frozen in liquid nitrogen, and stored at -80ºC.

- *HIS-Mcm7ΔC2.*

Essentially like HIS-Mcm7ΔC1. Except that after the Superdex™ 200 Increase 10/300 column, peak fractions were pooled, a 1:7 dilution with buffer E was made, and the protein was concentrated onto a MonoQ™ 5/50 GL column. The MonoQ was eluted with a 0.1-0.5M NaCl linear gradient in buffer F over 20CVs. Finally, Mcm7ΔC2 was aliquoted, snap-frozen in liquid nitrogen, and stored at -80ºC.

- *HIS-Cdt1 Flag.*

Like HIS-Mcm2 at the beginning of the process with some changes at the end. After the Superdex200 16/600 column, peak fractions were injected to a Superdex™200 Increase 10/300 column pre-equilibrated in buffer G. Peak fractions were pooled and concentrated with a 10K MWCO centricon. Protein was finally aliquoted, snap-frozen in liquid nitrogen, and stored at -80ºC.

- *HIS-Cdt1 S460, HIS-Cdt1 M471.*

Exactly like HIS-Mcm4 C-WHD, with the only exception that HIS-Cdt1 M471was concentered with a 10K MWCO centricon before being aliquoted.

- *HIS-Cdt1 S272, HIS-Cdt1 S272 1-1, HIS-Cdt1 1-1, and HIS-Cdt1 2-1.*

The starting cultures were 0.75L in volume for the Cdt1 S272 and S272 1-1, and 1.5L for the other two proteins. Essentially like the HIS-Mcm2 purification protocol, but with several changes at the end of the process. After the Ulp1 protease digestion, the protein was applied to a Superdex™ 200 Increase 10/300 pre-equilibrated in buffer D. Then, fractions containing the protein were pooled and injected to a second Superdex™ 200 Increase 10/300 pre-equilibrated in buffer G. Peak fractions of the second gel filtration column were aliquoted, snap-frozen in liquid nitrogen, and stored at -80ºC.

- *HIS-Cdt1 A495 and Cdt1 S436.*

To purify these proteins, we followed the protocol described by Frigola et al., 2017.

Formation of Mcm2-7+Cdt1 complexes from individually purified subunits.

We followed the protocol described by Frigola et al., 2013. The only change was that the gel filtration buffer also contained 5mM $Mg(OAc)_2$.

Supplementary Table 1. Oligonucleotides used in this study.

Primer	Sequence (5'→ 3')	Target	Direction
MG1	AGCTCTGATAATAGAAGACGTAG	*Mcm2*	FOR
MG2	TTATTAGTGACCCAAGGTATAAATTGC	*Mcm2*	REV
MG3	GGTGAAGGCTCAACGGGATTTGATG	*Mcm3*	FOR
MG4	CGATAAAGTTTGGAGAGTCTGAAGG	*Mcm3*	REV
MG5	AGCTCTCAACAGTCTAGCTCT	*Mcm4*	FOR
MG6	TCATCAGACACGGTTATTCA	*Mcm4*	REV
MG7	GGATCATTTGATAGACCGGAAATATACAG	*Mcm5*	FOR
MG8	CAGAAGTGGTGTATGAAGG	*Mcm5*	REV
MG9	CTGTTAGGATCCTCATCCCCTTTTCCAGCTG	*Mcm6*	FOR
MG10	ATATTACCCGGGTCATCCCCTTTTCCAGCTG	*Mcm6*	FOR
MG11	AGATATGGATCCTTATTAGCTGGAATCCTGTGG	*Mcm6*	REV
MG12	CCACAGGATTCCAGCTAATAACTCGAGATAT	*Mcm6*	REV
MG13	GGAAGTGCGGCACTTCCATCAATTCAG	*Mcm7*	FOR
MG14	TTAGGATCCAGTGGCACAGCTAATAGTAG	*Cdt1*	FOR
MG15	TTAGGATCCGATTATAAAGATGACGATGACAAGGATTATAAAGATGACGATGACAAGGATTATAAAGATGACGATGACAAGGGTGGATCAAGTGGCACAGCTAATAGTAG	*Flag Cdt1*	FOR
MG16	TCAAAACAACAAGATTGATAAGCGGCCGCT	*Cdt1*	REV
MG17	TAATTTCTCGAGTTATCAATCTTGTTGTTTTGA	*Cdt1*	REV
MG18	TCAATCTTGTTGTTTTGATTTGTG	*Cdt1*	REV
ARS305-F-PC-bio-Eco	GGTGTATGCATGCTACTGTTTGAATTCCCATTATCGAAGGCAC	*ARS305*	FOR
ARS305-R	CTCTAGCAAAAAGTCTAC	*ARS305*	REV
MG19	CAACAGACCCTAAAACCGGTTAA	*Mcm4ΔC-WHD*	REV
MG20	AGCGGTAAATCAGTTATTCAGAG	*Mcm4 C-WHD*	FOR
MG21	GTCTCAGGATCCAATTGGCTAA	*Mcm5ΔC-WHD*	REV
MG22	TTACATGGATCCTTACCCATCATCATTGTCATCATTG	*Mcm6ΔC-WHD*	REV
MG23	ATATTAGGATCCTCAGGTGTAATTACGAGTGAG	*Mcm6 C-WHD*	FOR
MG24	GGAATCATTGTATCAAGAAACCTAA	*Mcm7ΔC1*	REV
MG25	GGTAACACTTTGAAATTCGTGTAA	*Mcm7ΔC2*	REV
MG26	AATGTAGGATCCATGAGTAAAGGAGAGGGTAC	*Cdt1 S272*	FOR
MG27	GGTATGAGCTCTAATTCTAAAACAG	*Cdt1 S436*	FOR
MG28	TTATTTGGATCCTCAAGATATTTGGCGAATAG	*Cdt1 S460*	FOR
MG29	AATAAAGGATCCATGTACAAAGAGAAGATGC	*Cdt1 M471*	FOR

Supplementary Table 2. Plasmids used in this study.

**Plasmid**	**Cloning Vector**	**Insert**	**Reference**
pMG1	Champion^{TM} pET SUMO	*Mcm2*	This study
pMG2	Champion^{TM} pET SUMO	*Mcm3*	This study
pMG3	Champion^{TM} pET SUMO	*Mcm4*	This study
pMG4	Champion^{TM} pET SUMO	*Mcm5*	This study
pMG5	pET 28hisSUMO	*Mcm6*	This study
pET16b Mcm7	pET16b	*Mcm7*	Davey et al., 2003
pMG6	pET 28hisSUMO	*Flag Cdt1*	This study
pMG7	Champion^{TM} pET SUMO	*Mcm4ΔC-WHD* (1-2532bp)	This study
pMG8	Champion^{TM} pET SUMO	*Mcm4 C-WHD* (2557-2802bp)	This study
pMG9	Champion^{TM} pET SUMO	*Mcm5ΔC-WHD* (1-2097bp)	This study
pMG10	pCP6HP	*Mcm6ΔC-WHD* (1-2610bp)	This study
pMG11	pET 28hisSUMO	*Mcm6 C-WHD* (2614-3054bp)	This study
pMG12	pET 28hisSUMO	*Mcm6 C-WHD 1-1*: E945A, D947A, L951A, E953A, and Y954A (2614-3054bp)	This study
pMG13	Champion^{TM} pET SUMO	*Mcm7ΔC1* (1-2196bp)	This study
pMG14	Champion^{TM} pET SUMO	*Mcm7ΔC2* (1-2415bp)	This study
pETS CMC	pET 28hisSUMO	*Cdt1 S272* (814-1815bp)	Frigola et al., 2017
pMG15	pET 28hisSUMO	*Cdt1 S460* (1378-1815bp)	This study
pMG16	pET 28hisSUMO	*Cdt1 M471* (1411-1815bp)	This study
ARS 305-BP	pBR322	*ARS305*	Tanaka and Araki, 2010
pMG17	pET28hisSUMO	*Cdt1 1-1*: R486A L487A, and R490A	Li et al., 2012
P11-AM3	pGEX6p1	*Cdc6*	Frigola et al., 2013
pETS CC1	pET28hisSUMO	*Cdt1 A495* (1483-1815bp)	Frigola et al., 2017
pMG18	Champion^{TM} pET SUMO	*Cdt1 S436* (1306-1815bp)	This study
pMG19	pET28hisSUMO	*Cdt1 S272 1-1*: R486A L487A, and R490A (814-1815bp)	This study
pMG20	pET28hisSUMO	*Cdt1 2-1*: M471A, Y472A, and K475A	This study
pMG21	pET28hisSUMO	*Mcm6 1-1*: E945A, D947A, L951A, E953A, and Y954A	Li et al., 2012
pMG22	pET28hisSUMO	*Mcm6 2-1*: Y906F, D971N, and R972E	This study
pMG23	pET28hisSUMO	*Mcm6 2-2*:	This study

		Y906K, V910D, N914E, A921F, and R925D	
pMG24	pET28hisSUMO	*Mcm6 2-3*:<br>Y906R, N914Y, and A921Y	This study
pMG25	pET28hisSUMO	*Mcm6 2-4*:<br>Y906R, V910D, N914Y, R918E, A921Y, and R925E	This study
pMG26	pET28hisSUMO	*Mcm6 2-5*:<br>D924N, M975Q, and I977E	This study
pMG27	pET28hisSUMO	*Mcm6 2-6*:<br>Y906F, D924N, R972E, and M975Q	This study

Supplementary Table 3. Comparison between wild type and mutated proteins used in this study.

Protein	Length (aa)	Description	Reference
Mcm2wt	868	wt	This study
Mcm3wt	971	wt	This study
Mcm4wt	933	wt	This study
Mcm4ΔC	843 (1-843)	Deletion of C-WHD	This study
Mcm4 C-WHD	80 (853-933)	C-WHD	This study
Mcm5wt	775	wt	This study
Mcm5ΔC	698 (1-698)	Deletion of C-WHD	This study
Mcm6	1017	wt	This study
Mcm6ΔC	869 (1-869)	Deletion of C-WHD	This study
Mcm6 C-WHD	146 (872-1017)	C-WHD	This study
Mcm6 C-WHD 1-1	146 (872-1017)	C-WHD 1-1: E945A, D947A, L951A, E953A, and Y954A	This study
Mcm6 1-1	1017	E945A, D947A, L951A, E953A, and Y954A	Li et al., 2012
Mcm6 2-1	1017	Y906F, D971N, and R972E	This study
Mcm6 2-2	1017	Y906K, V910D, N914E, A921F, and R925D	This study
Mcm6 2-3	1017	Y906R, N914Y, and A921Y	This study
Mcm6 2-4	1017	Y906R, V910D, N914Y, R918E, A921Y, and R925E	This study
Mcm6 2-5	1017	D924N, M975Q, and I977E	This study
Mcm6 2-6	1017	Y906F, D924N, R972E, and M975Q	This study
Mcm7wt	845	wt	This study
Mcm7ΔC1	731 (1-731)	Deletion of C-WHD	This study
Mcm7ΔC2	804 (1-804)	Deletion of specific *S. cerevisiae* C terminal (last 41 aa)	This study
Cdt1wt	604	wt	This study
Cdt1 S272	333 (272-604)	Deletion of N' terminal domain (NTD)	Frigola et al., 2017
Cdt1 S272 1-1	333 (272-604)	Deletion of NTD: R486A L487A, and R490A	This study
Cdt1 S436	169 (436-604)	Deletion of NTD and middle WHD (M-WHD), maintaining	This study

		loop interacting with Mcm2 and Mcm6	
Cdt1 S460	145 (460-604)	Deletion of NTD and M-WHD, maintaining loop interacting with Mcm6	This study
Cdt1 M471	134 (471-604)	Deletion of NTD and M-WHD, maintaining loop interacting with Mcm6 C-WHD	This study
Cdt1 A495	110 (495-604)	Deletion of NTD, M-WHD and loop between M- and C-WHD	Frigola et al., 2017
Cdt1 1-1	604	R486A L487A, and R490A	Li et al., 2012
Cdt1 2-1	604	M471A, Y472A, and K475A	This study

Guerrero-Puigdevall et al. Supplementary Figure 1

a

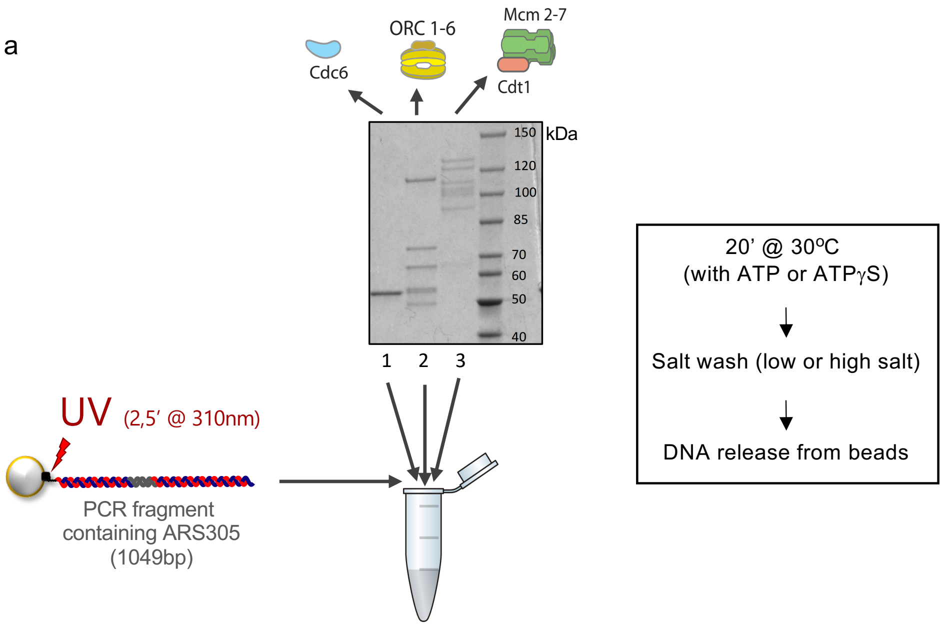

b

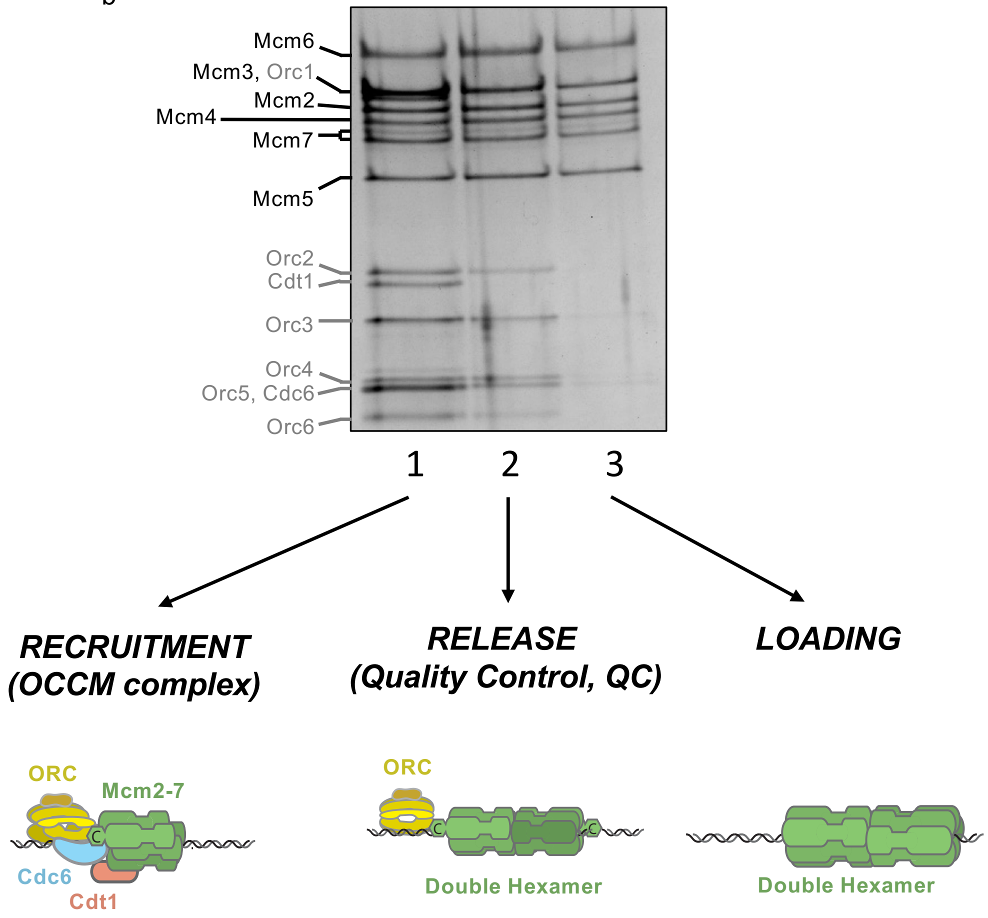

TABLE 1. Different outcomes of the licensing assay with purified proteins.

NAME IN THE ASSAY	***ATP SOURCE USED***	***SALT WASH AFTER PROTEIN INCUBATION***	***EXPECTED PROTEINS ON DNA AFTER WASHES***
**RECRUITMENT**	ATPγS *(slowly hydrolysed analogue of ATP)*	Low salt *(0.3M Potassium Acetate)*	ORC1-6, Cdt1, Cdc6 & Mcm2-7
**RELEASE**	ATP	Low salt *(0.3M Potassium Acetate)*	ORC1-6 & Double hexamer
**LOADING**	ATP	High salt *(0.5M Sodium Cloride)*	Double hexamer

Supplementary Figure 1. PreRC assembly with purified proteins.

(a) Outline of the preRC assembly using purified proteins. ORC1-6 complex is purified as a whole complex (lane 2), Cdc6 as an individual protein (lane 1) and MCM-Cdt1 either as a complex (lane 3) or from individual subunits. A PCR fragment containing ARS305 (length, 1049 bp) was amplified using a primer containing a photocleavable biotin. Magnetic beads coated with streptavidin are coupled to PCR products containing ARS305. All the proteins, together with the magnetic beads coupled to origin DNA, are tested in a tube assay for licensing capacity. The presence of the photocleavable biotin, allows the release of the DNA from the beads, after a short irradiation of 2,5 minutes at 310nm. The variables used in this assay are: ATP source in the reaction (ATP or ATPgS) and salt washes after the licensing reaction (low or high salt wash). Overall, different combinations of these variables lead to three different outcomes, named: recruitment, release and loading. A summary of the different conditions for these three outcomes is shown in table 1. (b) An example of the different outcomes described in table 1. Under recruitment conditions (lane 1), all preRC members are bound to origin DNA assembling the OCCM complex. Release conditions retain only the ORC and double hexamer complexes (lane 2). If the criteria for double hexamer formation is not met, only ORC will remain bound to origin DNA and the MCM-Cdt1 will be released by an ATPase dependent quality control (QC). Finally, under loading conditions, only double hexamers are stable enough to remain on the DNA after the high salt wash (lane 3).

Guerrero-Puigdevall et al. Supplementary Figure 2

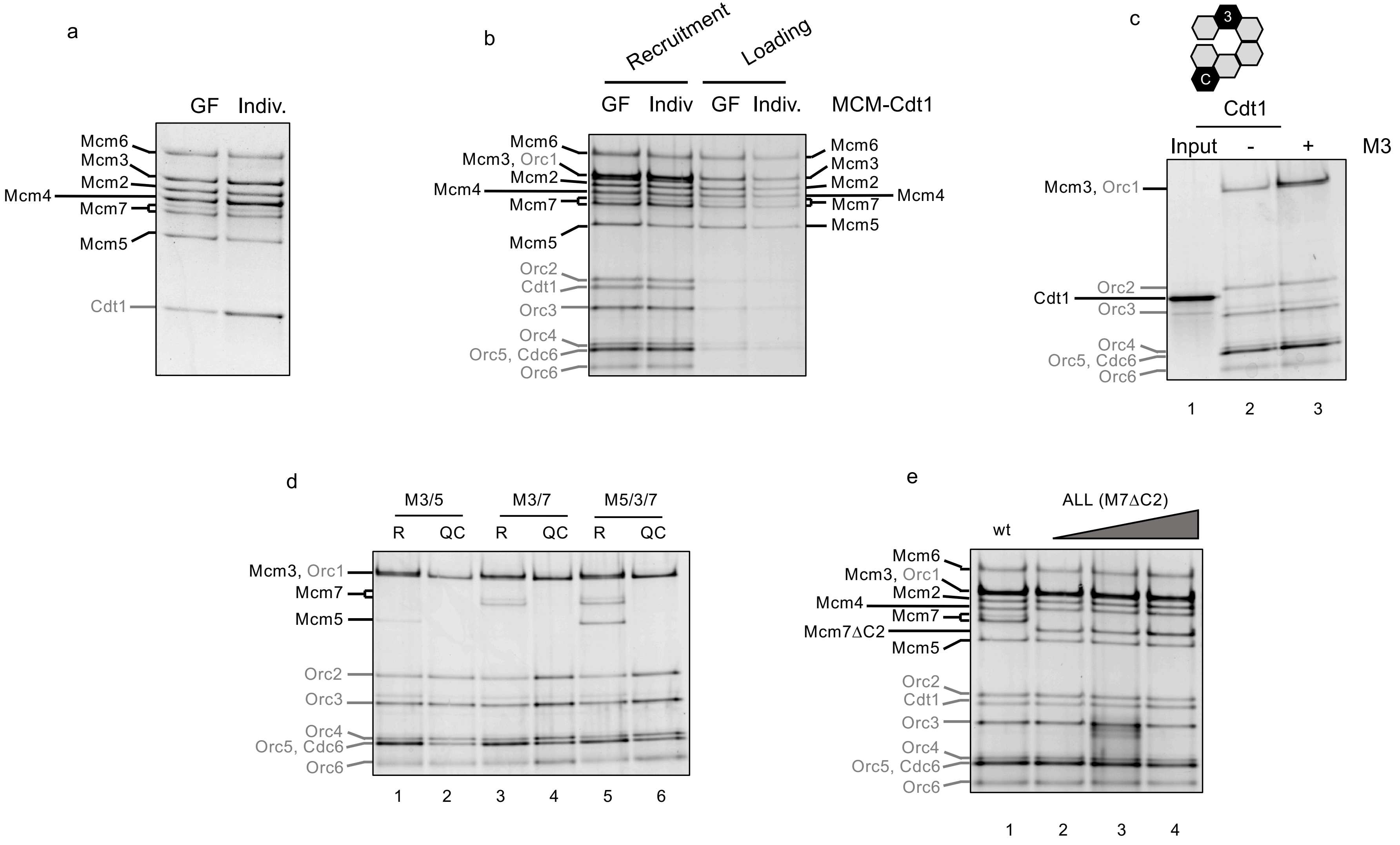

Supplementary Figure 2. Mcm5/3/7 recruitment.

(a) Coomassie staining of two different MCM-Cdt1 complexes used in the preRC assay; one isolated from gel filtration (GF) and a second one, where individual subunits were added straight into the assay (Indiv.). (b) Recruitment and loading of the MCM-Cdt1 complexes shown in a. (c) Recruitment of Cdt1 by OC, in minus or plus Mcm3. 20% of the Cdt1 used in the recruitment is shown in the input lane (lane1). (d) Recruitment conditions of the dimers Mcm3/5, Mcm3/7 and the trimer Mcm5/3/7 are shown in lanes 1,3 and 5 respectively (R). Release or quality control conditions of the same subcomplexes are shown in lanes 2, 4 and 6 (QC). (e) Recruitment of MCM-Cdt1, using either Mcm7 wt (lane 1) or increasing amounts of small C terminal deletion M7ΔC2 (last 41 amino acids, lanes 2-4). Note that the residues deleted are only present in *S. cerevisiae*.

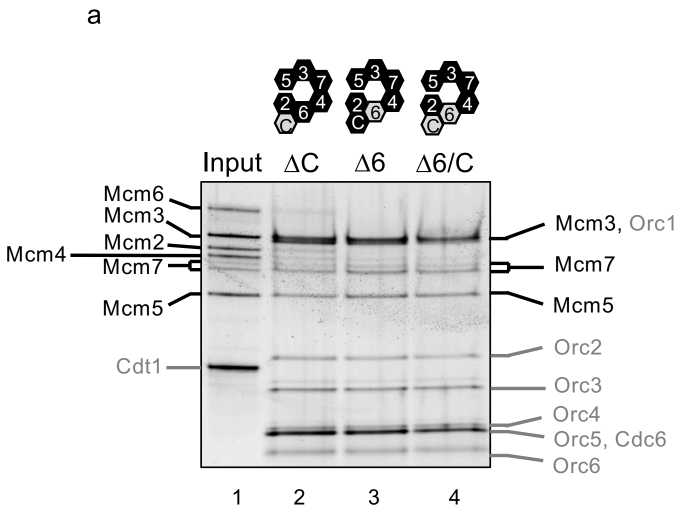

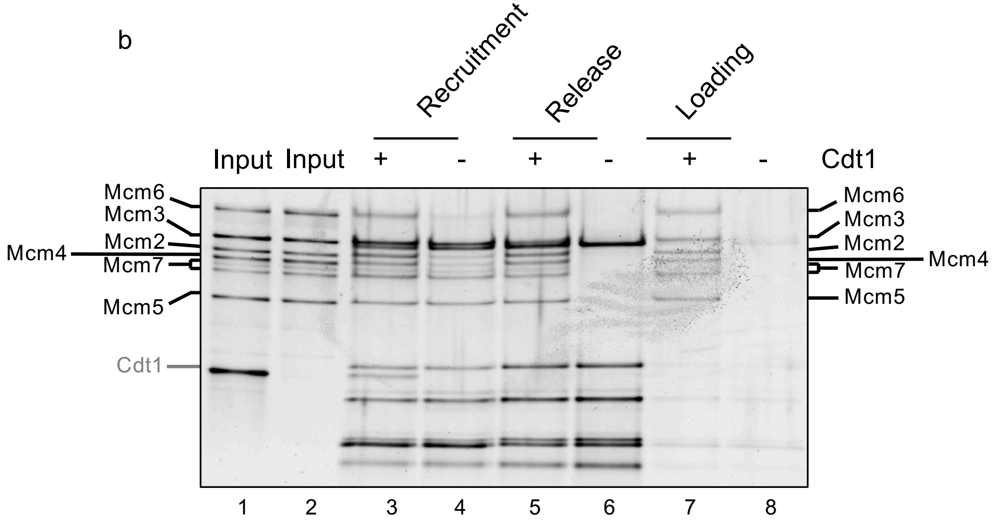

Supplementary Figure 3. Cdt1 is essential for MCM recruitment.

(a) Different combinations where proteins are omitted (grey hexamers). Note that the sub stochiometric amount of Mcm2 recruited in minus Cdt1 depends on the presence of its neighbouring subunit, Mcm6 (compare Mcm2 in lane 2 and 3). (b) PreRC assay of the MCM complex with the presence (+) or absence (-) of Cdt1. 20% of the used complexes are shown in the input lanes. The sub stochiometric MCM complex recruited in minus Cdt1 (recruitment, lane 4), cannot form DHs (loading, lane 8) and it is released from DNA by the quality control (lane 6).

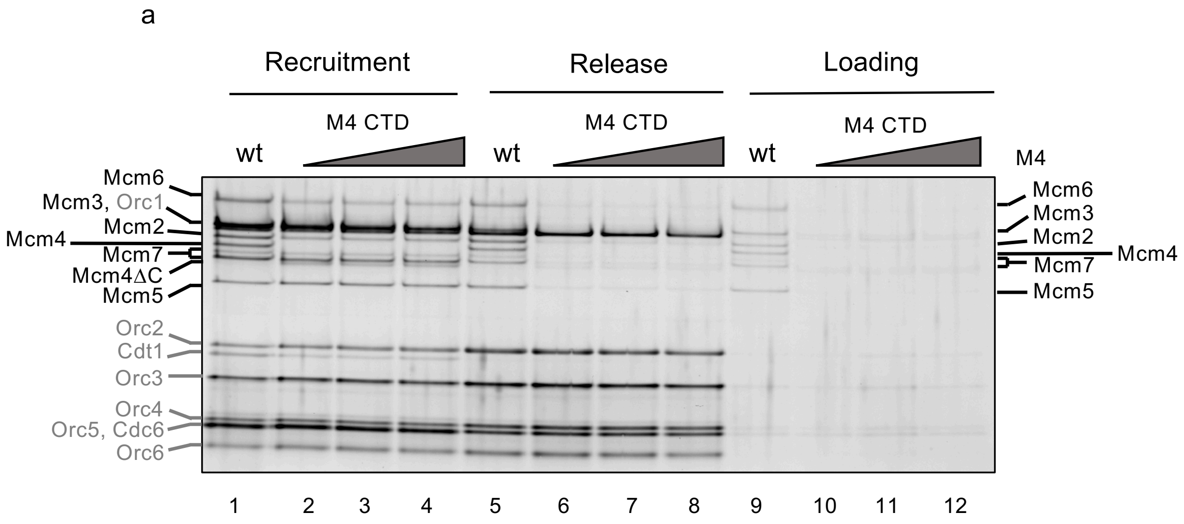
a
Recruitment
Release
Loading
wt
M4 CTD
wt
M4 CTD
wt
M4 CTD
M4
Mcm6
Mcm3, Orc1
Mcm2
Mcm4
Mcm7
Mcm4ΔC
Mcm5
Orc2
Cdt1
Orc3
Orc4
Orc5, Cdc6
Orc6
Mcm6
Mcm3
Mcm2
Mcm4
Mcm7
Mcm5
1 2 3 4 5 6 7 8 9 10 11 12

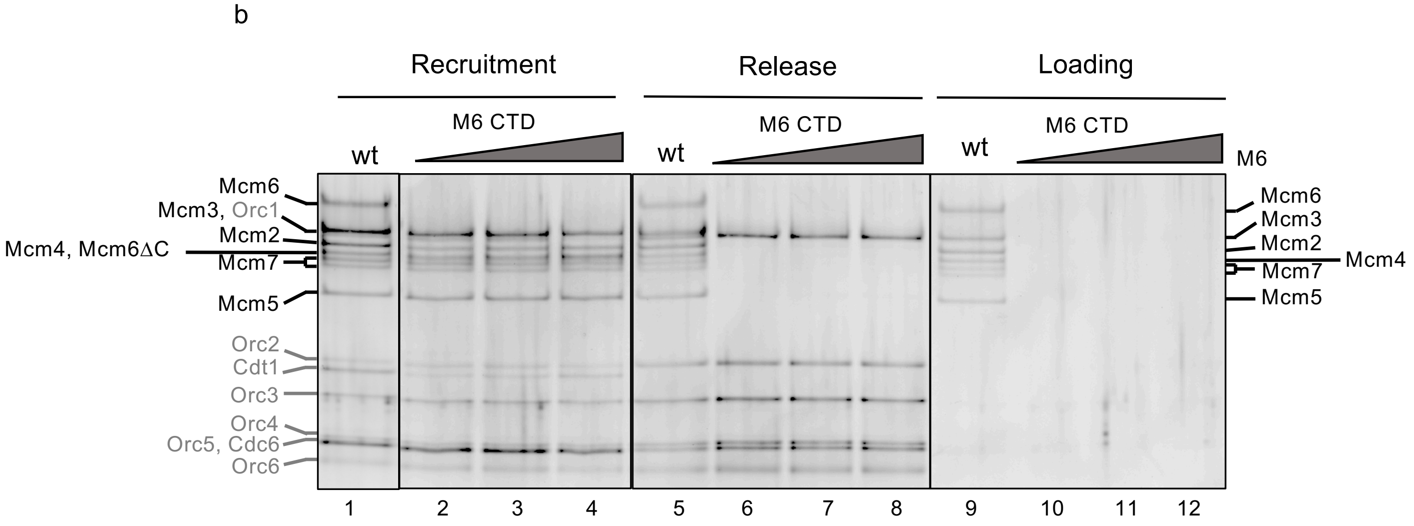
b
Recruitment
Release
Loading
wt
M6 CTD
wt
M6 CTD
wt
M6 CTD
M6
Mcm6
Mcm3, Orc1
Mcm2
Mcm4, Mcm6ΔC
Mcm7
Mcm5
Orc2
Cdt1
Orc3
Orc4
Orc5, Cdc6
Orc6
Mcm6
Mcm3
Mcm2
Mcm4
Mcm7
Mcm5
1 2 3 4 5 6 7 8 9 10 11 12

Supplementary Figure 4. The addition of the C-WHDs of Mcm4 and 6 separately, cannot rescue the defect shown by its deletion.

(a) preRC assay with MCM-Cdt1 containing all the wt proteins (wt) or Mcm4 delta C-WHD that has been supplemented by the addition of increasing amounts of the Mcm4 C-WHD (M4 CTD). The complementation of the MCM-Cdt1 complex, containing an Mcm4 delta C-WHD, with increasing amounts of the M4 C-WHD, does not improve either the recruitment (lanes 2-4) or the loading (lanes 10-12). (b) the same experiment than a, but using an MCM-Cdt1 complex containing an Mcm6 delta C-WHD, and complemented with the addition of Mcm6 C-WHD separately.

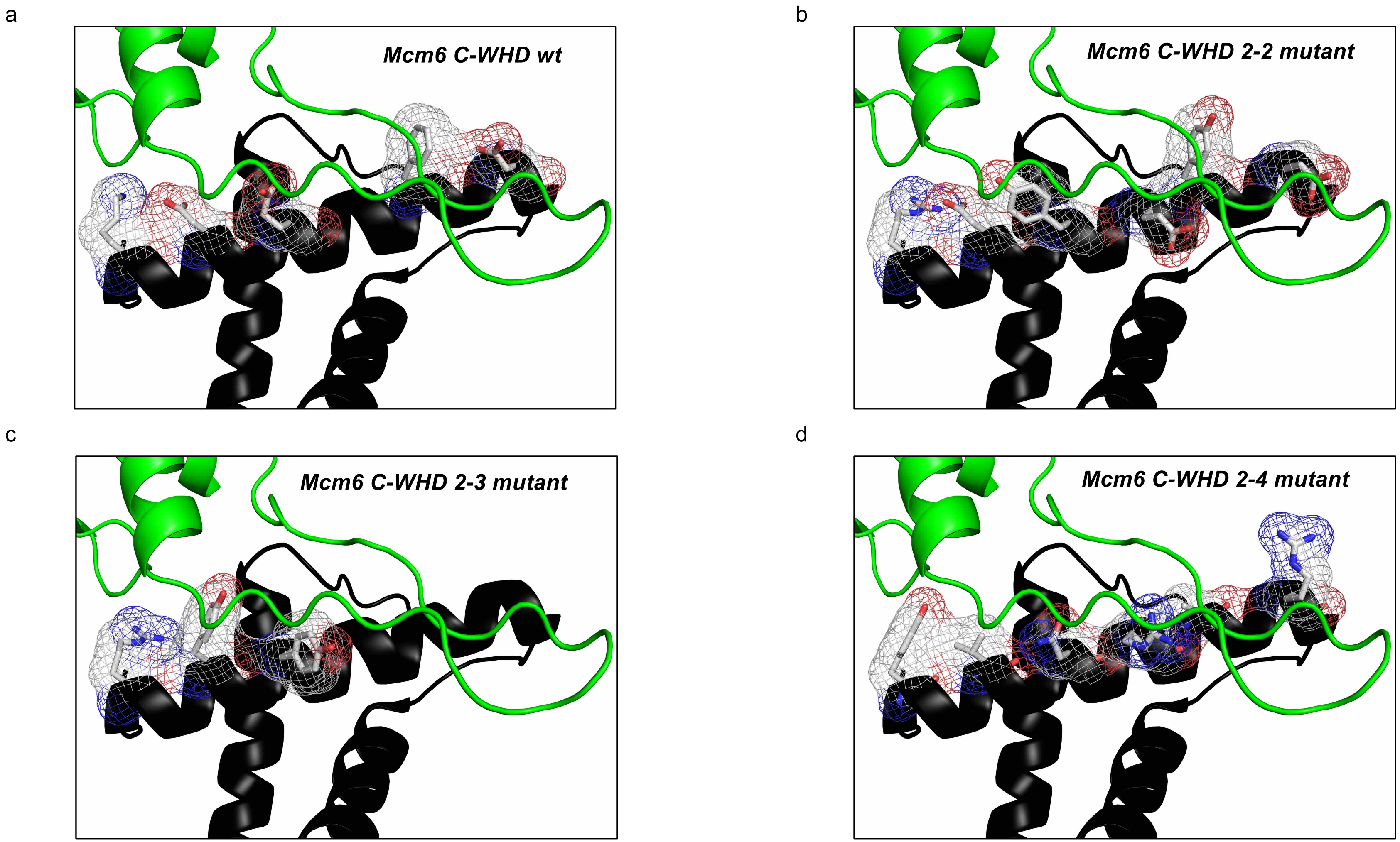
a
Mcm6 C-WHD wt
b
Mcm6 C-WHD 2-2 mutant
c
Mcm6 C-WHD 2-3 mutant
d
Mcm6 C-WHD 2-4 mutant

Supplementary Figure 5. Description of the Mcm6 2-2, 2-3 and 2-4 mutations.

Description of the different Mcm6 C-WHD mutations design to interfere with the interaction between Mcm6 C-WHD (black) and the Orc4 loop (green). (a) wt residues present in Mcm6 C-WHD: Y906, V910, N914, R918, A921 and R925. (b) Mcm6 2-2 mutation corresponding to semi-conserved replacement along the central helix in Mcm6: Y906K, V910D, N914E, A921F and R925D. (c) Mcm6 2-3 mutation corresponding to more disruptive replacement in the first half of the helix: Y906R, N914Y and A921Y and (d) Mcm6 2-4 mutation corresponding to severe substitutions along the central helix of Mcm6: Y906R, V910D, N914Y, R918E, A921Y and R925E. Side chains are depicted both in stick and mesh representation to account for the atomic volume of the residues.

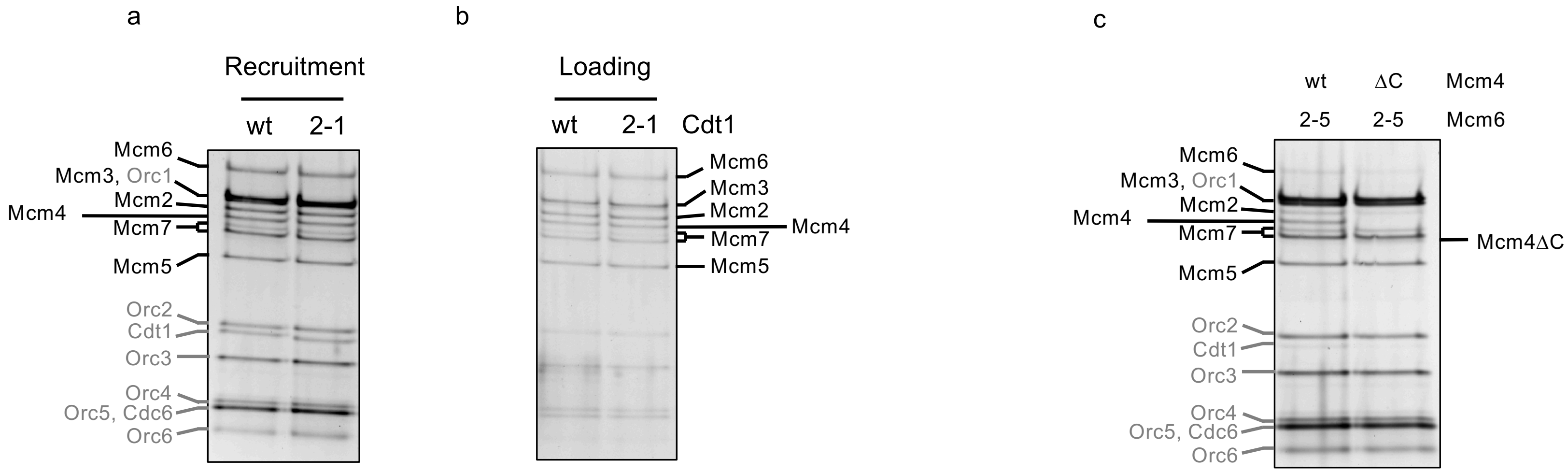

Supplementary Figure 6. PreRC assembly by Cdt1 2-1 mutation.

Recruitment and loading of the Cdt1 2-1 mutation are depicted in a and b respectively. (c) Recruitment studies of MCM-Cdt1 containing Mcm6 2-5 mutation and Mcm4 either wild type (wt) or C-WHD deleted (ΔC).

Guerrero-Puigdevall et al. Supplementary Figure 7

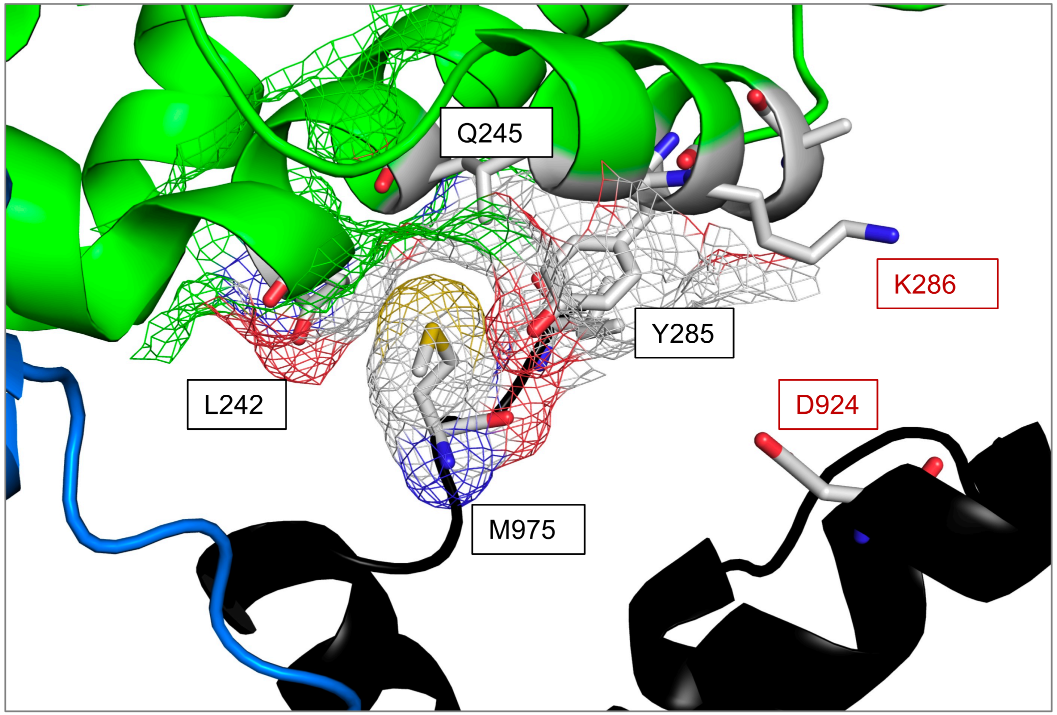

Supplementary Figure 7. Structural detailed description of the key residues involved in the Mcm6 C-WHD and Orc5 AAA+lid.

Orc5 is shown in green and Mcm6 in black. These two proteins interact via an ionic interaction mediated by Mcm6 D924 and Orc5 K286 (in red boxes). In addition, Mcm6 M975 interacts with a hydrophobic patch present at Orc5 and integrated by L242, Q245 and Y285 (in black boxes). Both of these residues in Mcm6 (D924 and M975) are changed in Mcm6 2-5 and 2-6 mutations. Side chains are depicted both in stick and mesh representation to account for the atomic volume of the residues.
