# Supplementary material for: Stabilisation of half MCM ring by Cdt1 during DNA insertion
Source: Nat Commun. 2021 Mar 19;12:1746. doi: 10.1038/s41467-021-21932-8 (PMC7979726; doi:10.1038/s41467-021-21932-8)
Supplement: Supplementary file 3 — Source Data [file 41467_2021_21932_MOESM3_ESM.pdf]

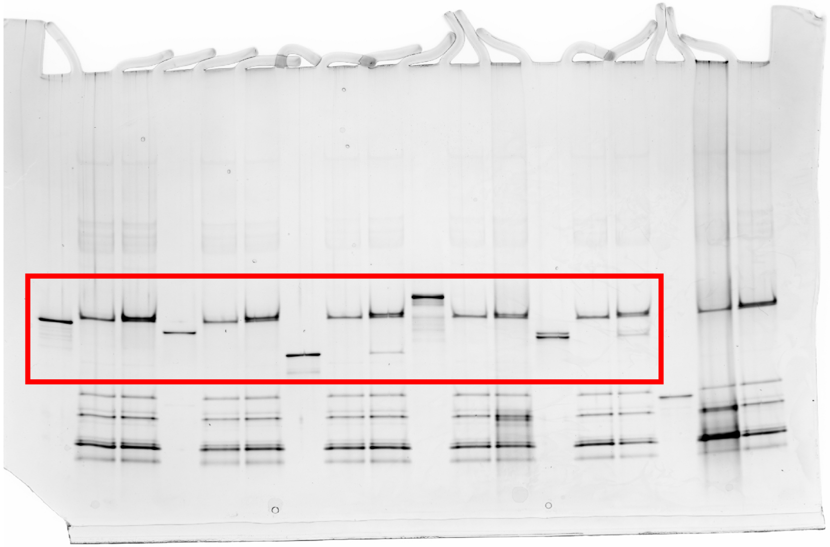

Fig. 1a

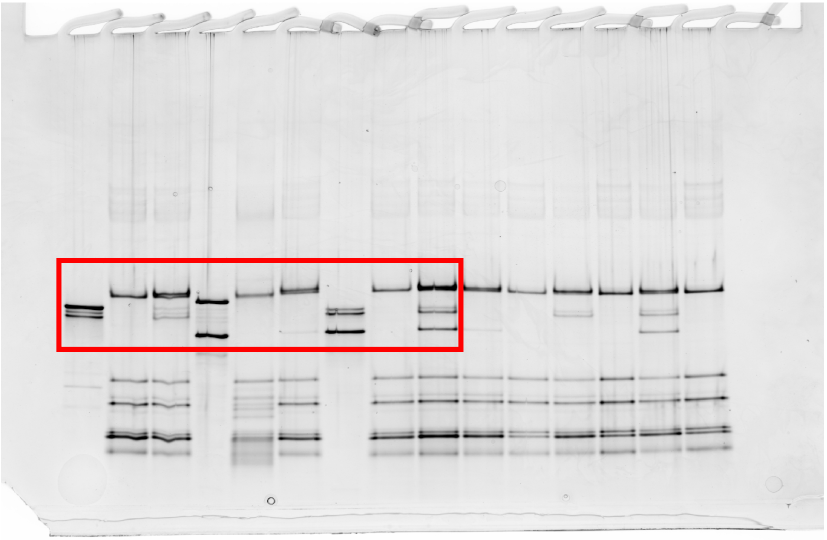

Fig. 1b

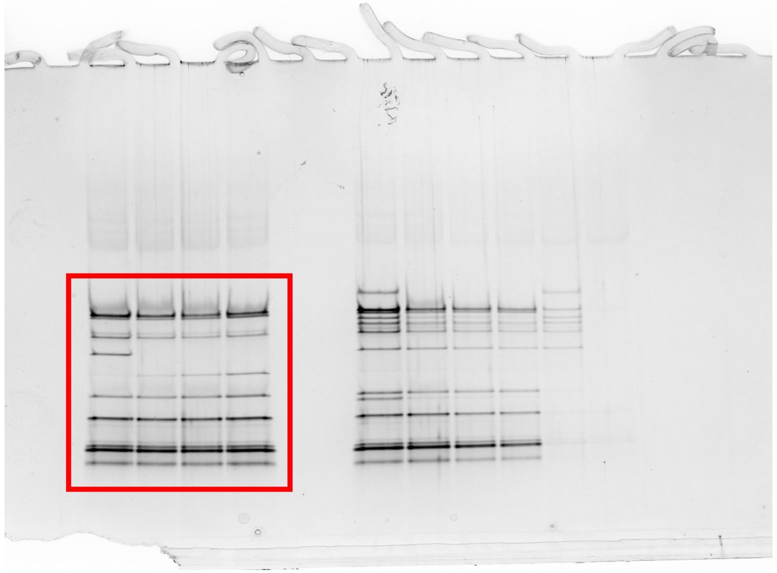

Fig. 1c

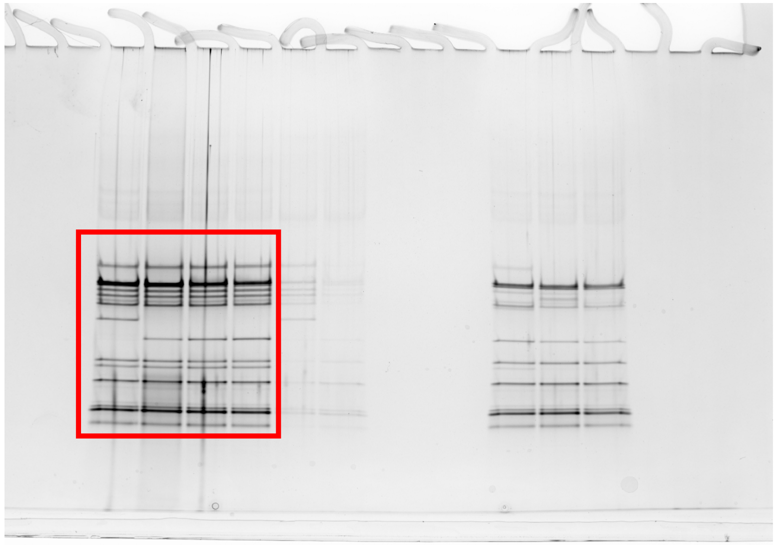

Fig. 1d

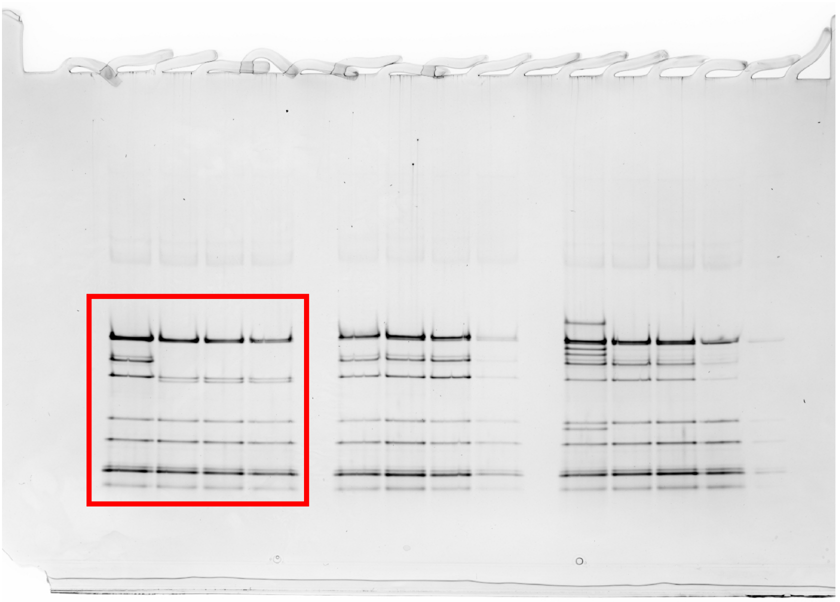

Fig. 1e

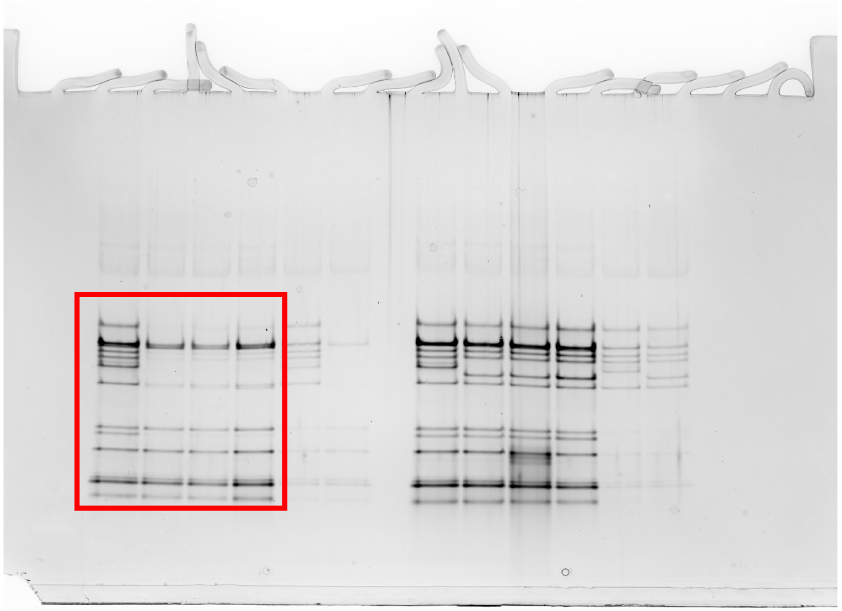

Fig. 1f

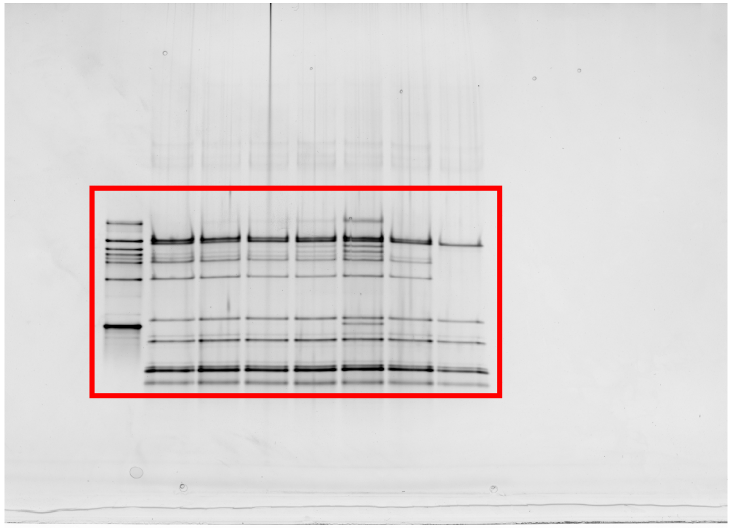

Fig. 2a

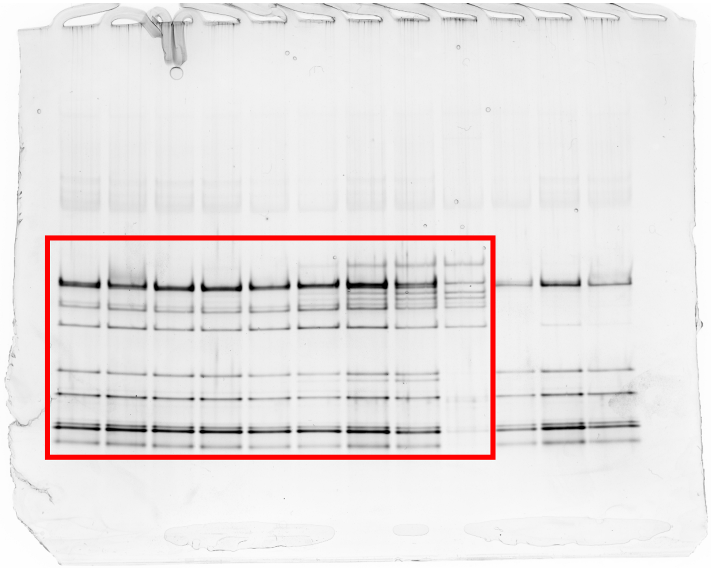

Fig. 2b

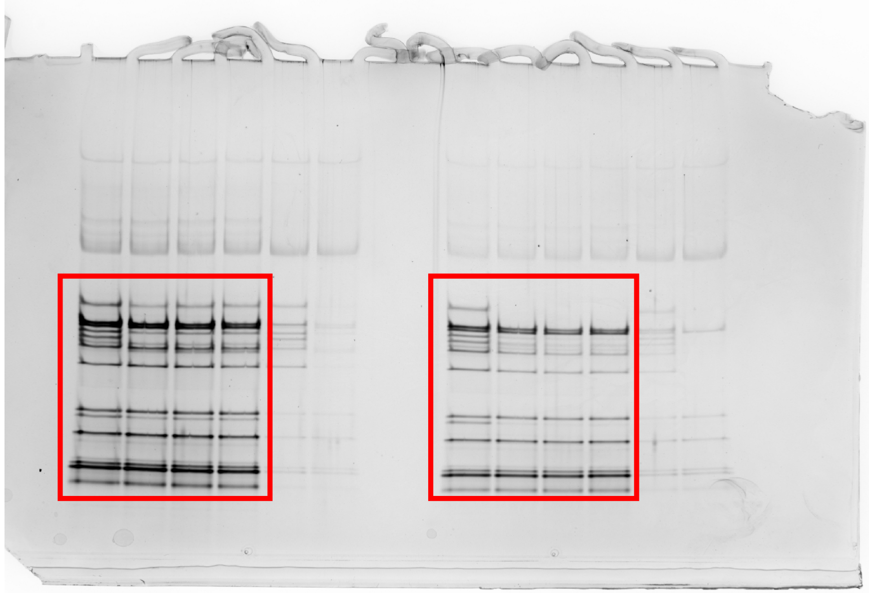

Fig. 2c

Fig. 2d

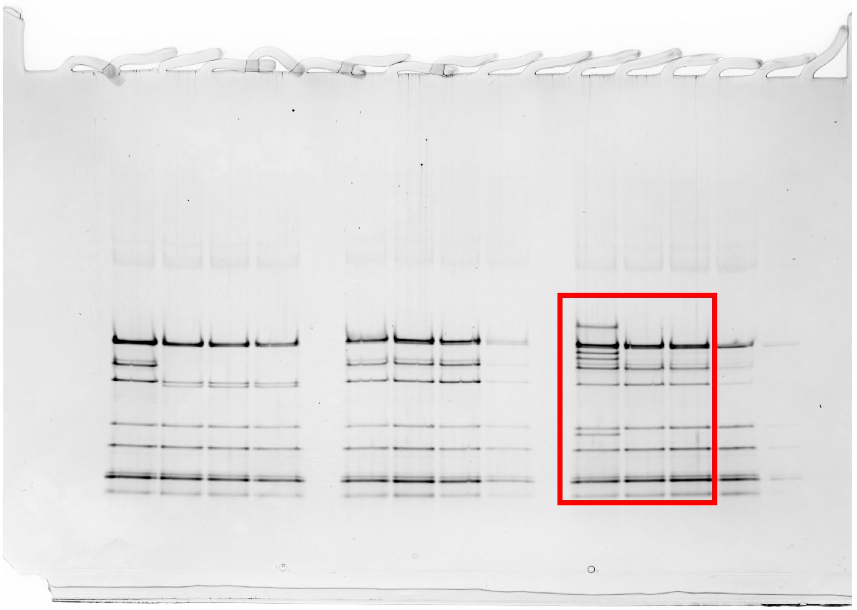

Fig. 2e

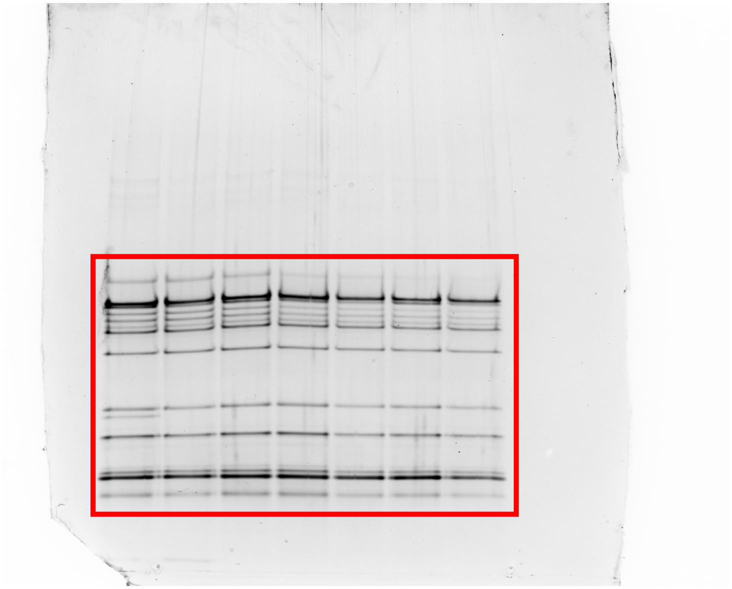

Fig. 3b

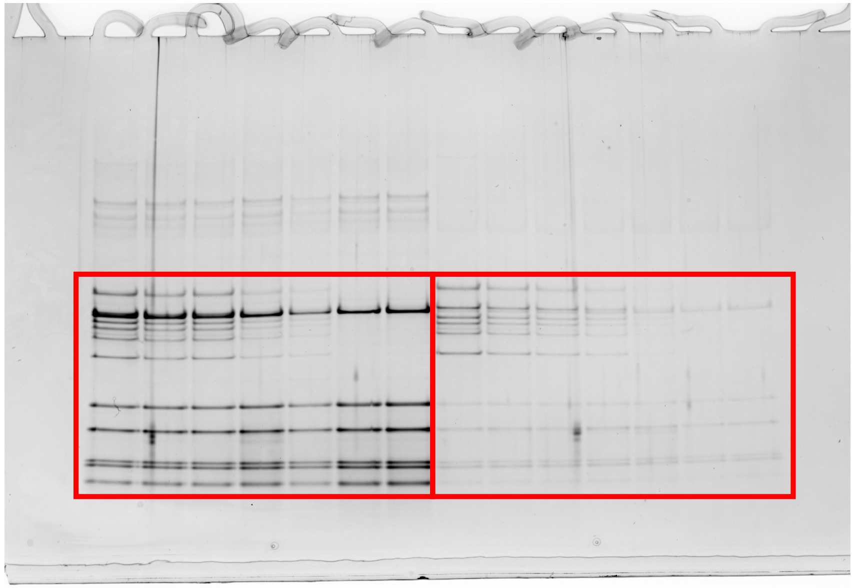

Fig. 3c Fig. 3d

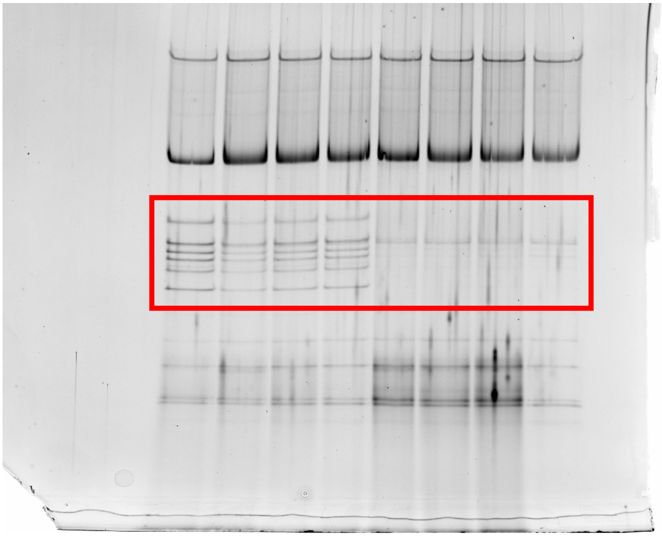

Fig. 3e

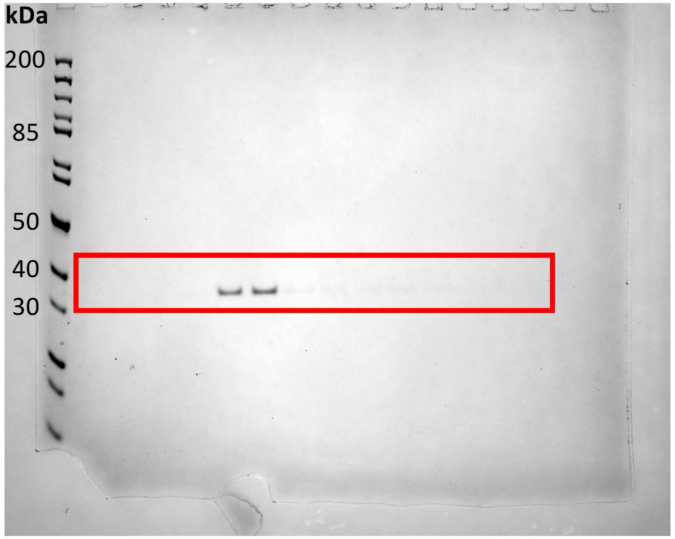

Fig. 4b (Cdt1 S272 wt)

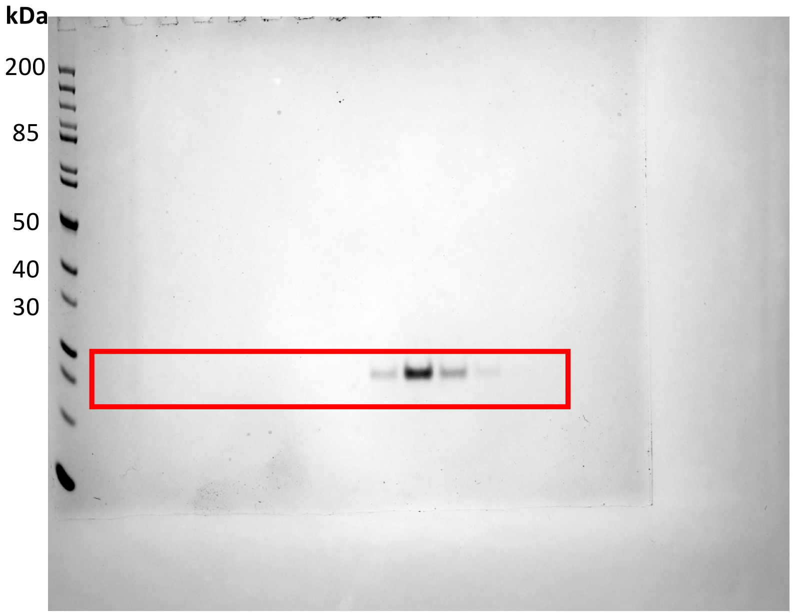

Fig. 4b (M6 C-WHD wt)

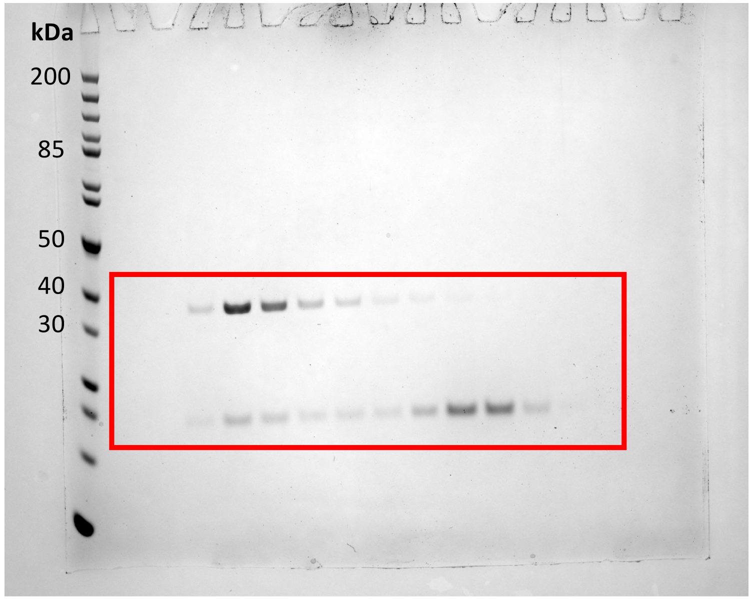

Fig. 4b. (Cdt1 S272wt and M6 C-WHD wt)

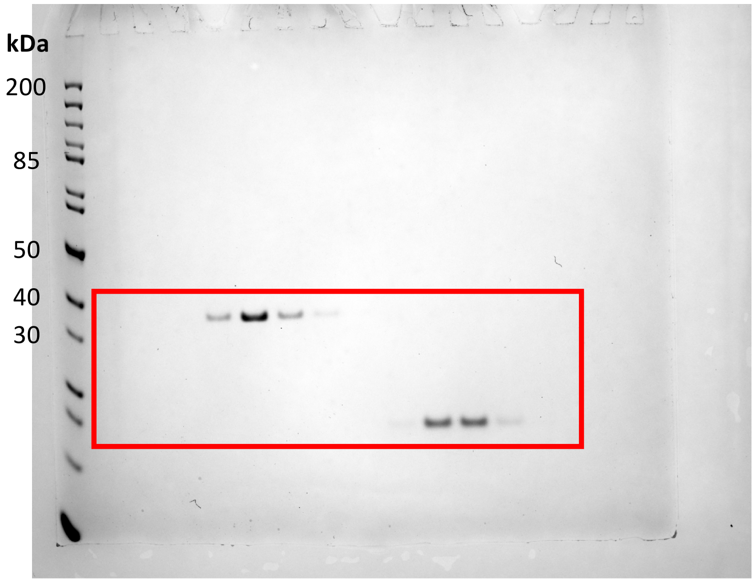

Fig. 4b. (Cdt1 S272 1-1 and M6 C-WHD wt)

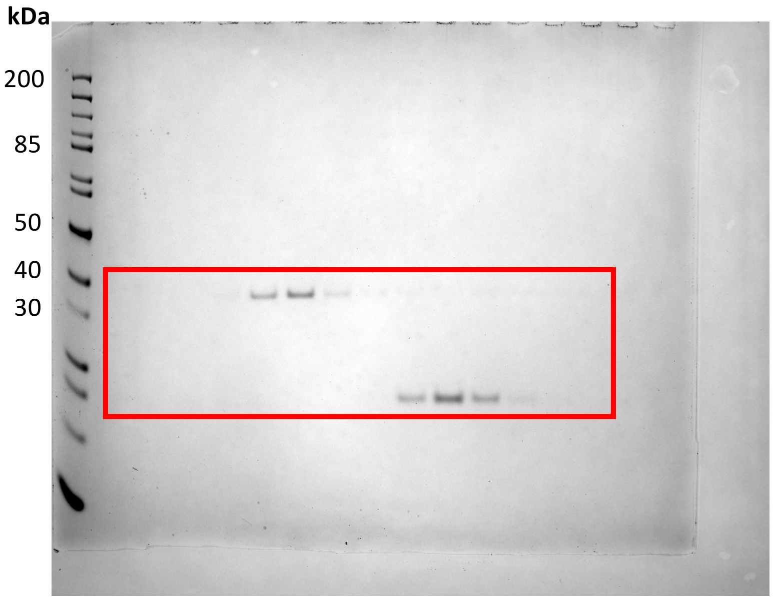

Fig. 4b. (Cdt1 S272wt and M6 C-WHD 1-1)

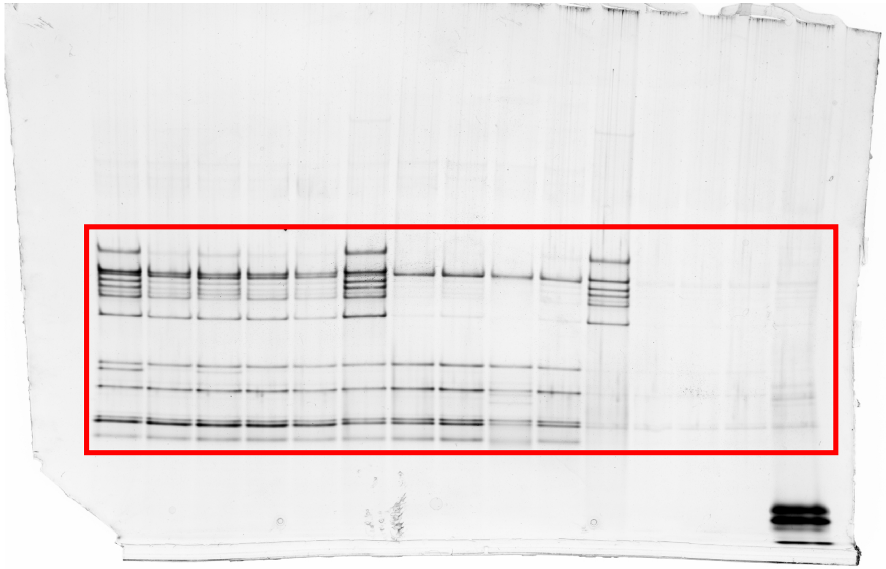

Fig. 4c

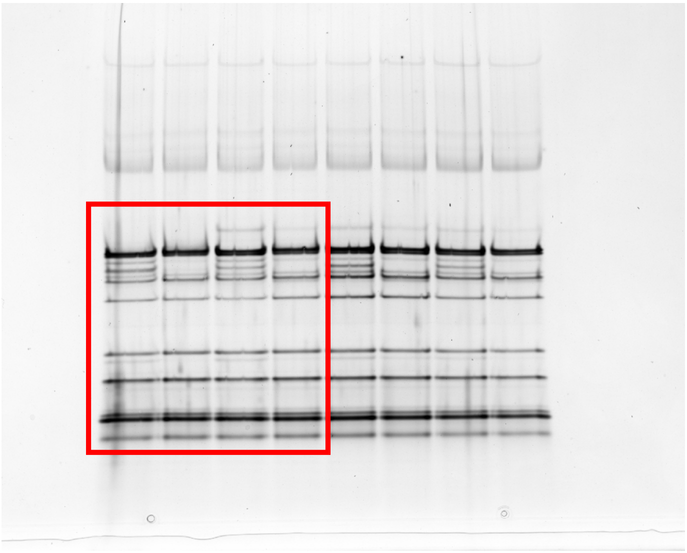

Fig. 4d

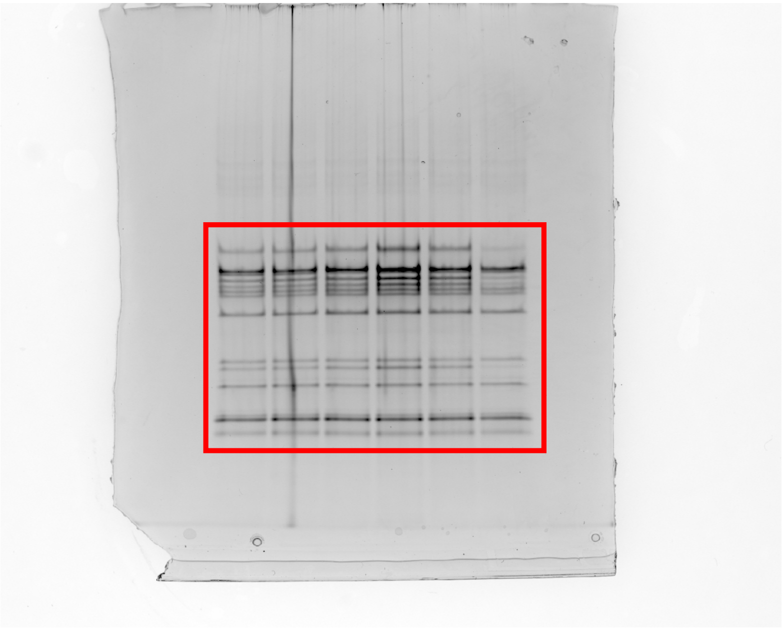

Fig. 5b

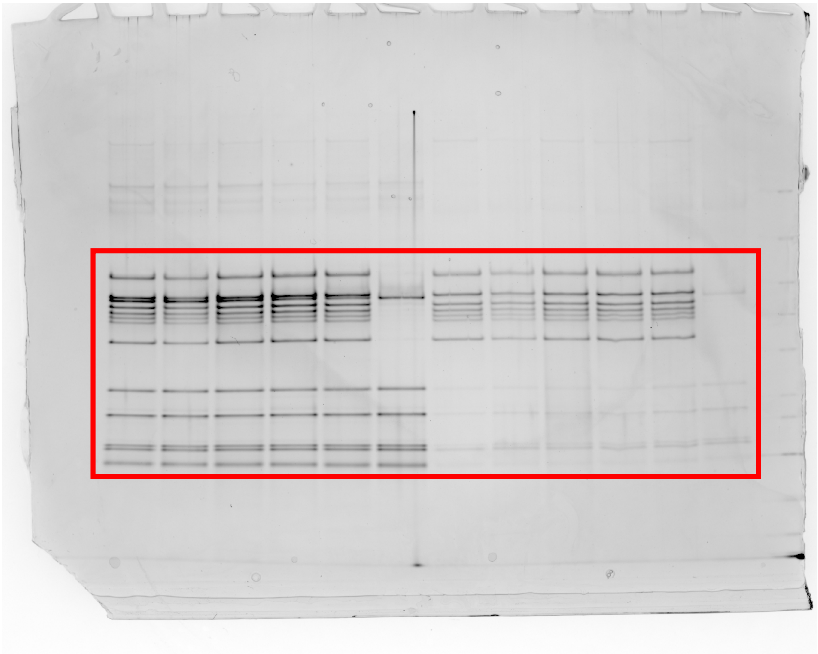

Fig. 5c

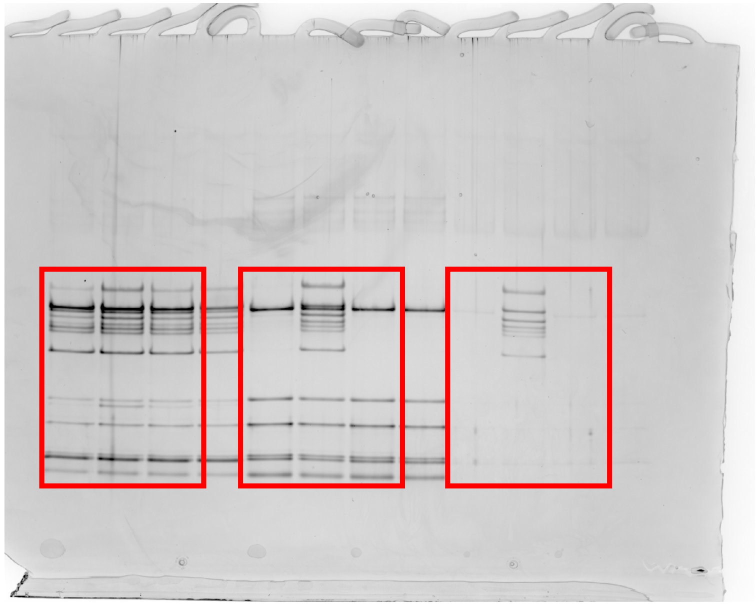

Fig. 6b

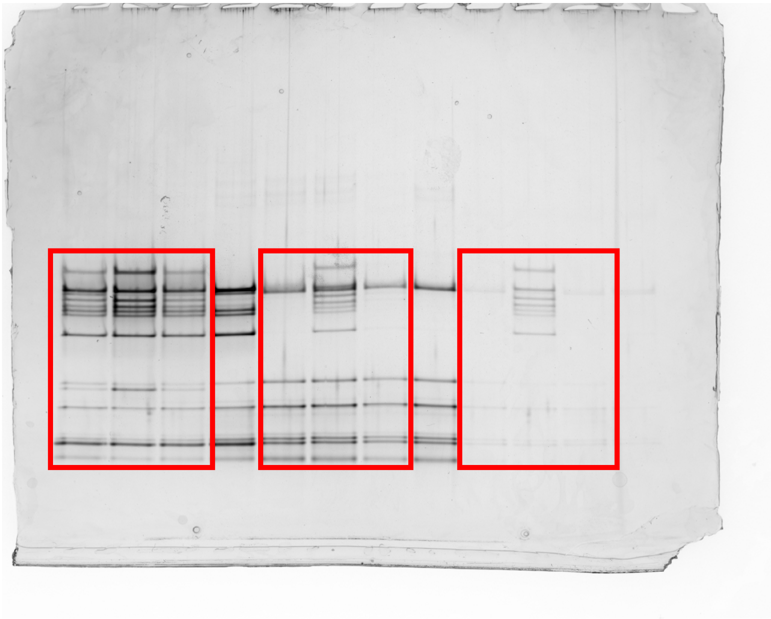

Fig. 6c

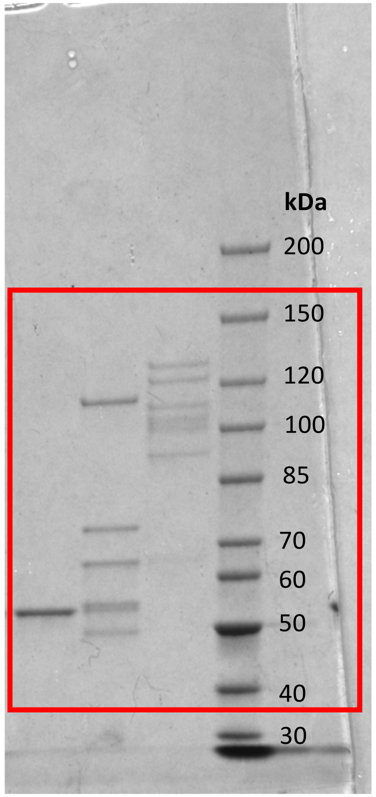

Supp. Fig. 1a

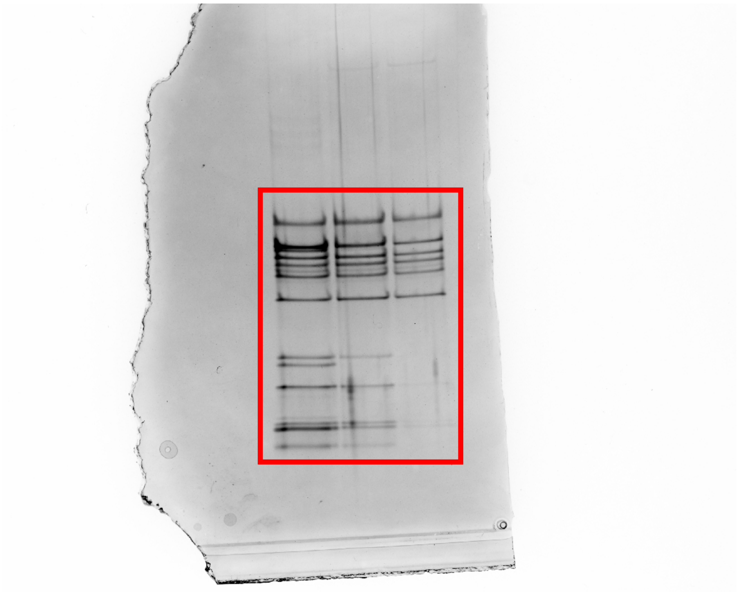

Supp. Fig. 1b

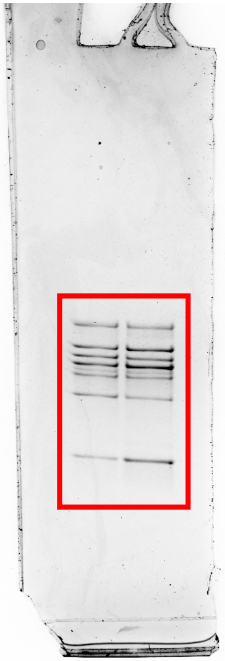

Supp. Fig. 2a

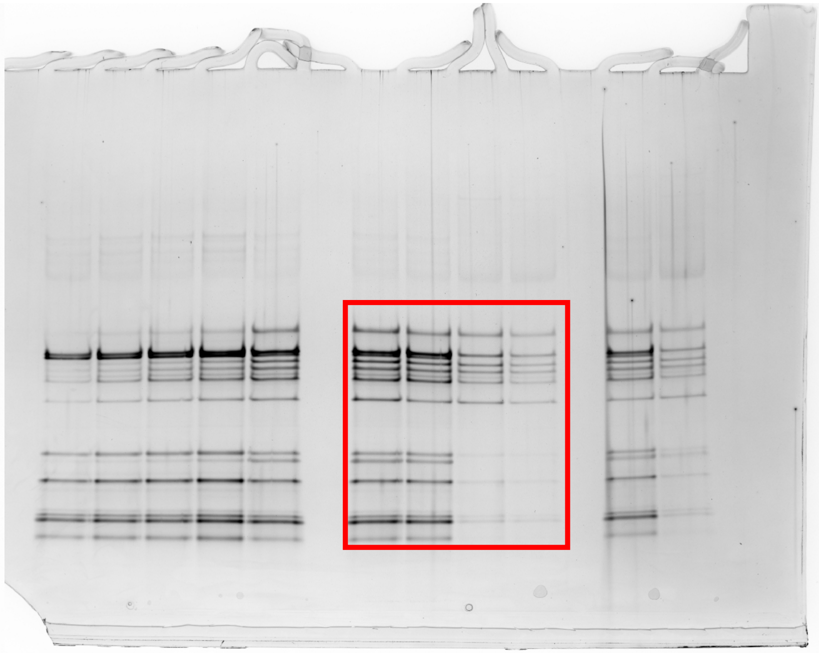

Supp. Fig. 2b

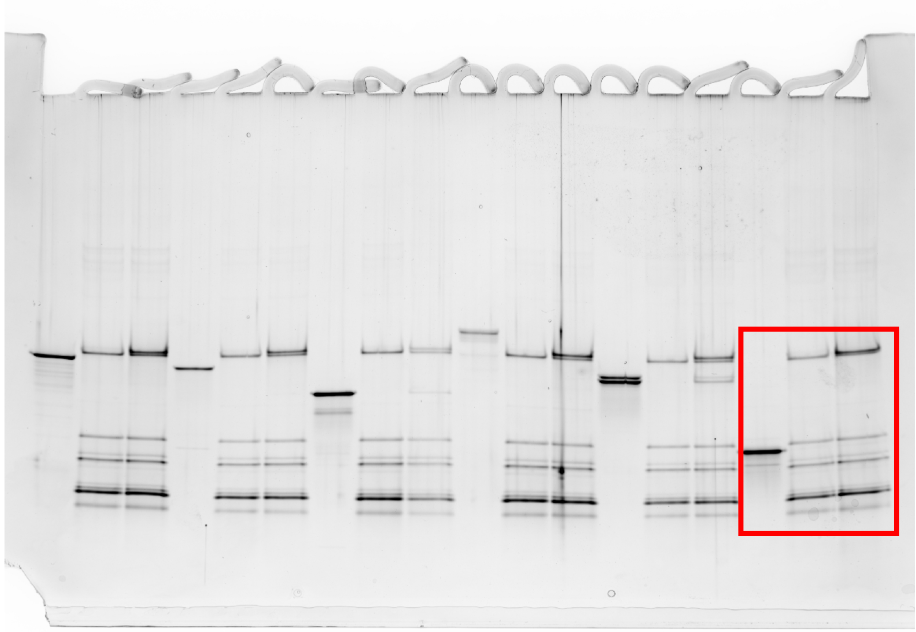

Supp. Fig. 2c

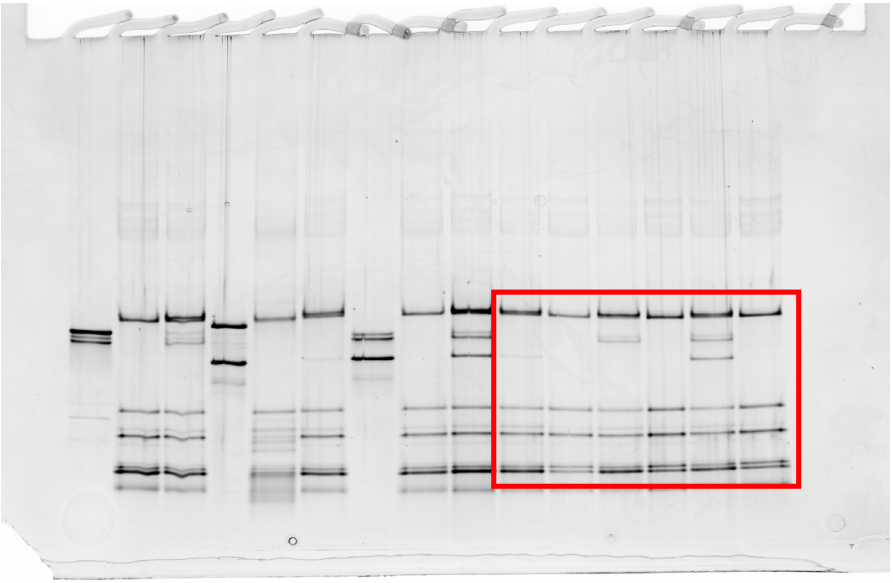

Supp. Fig. 2d

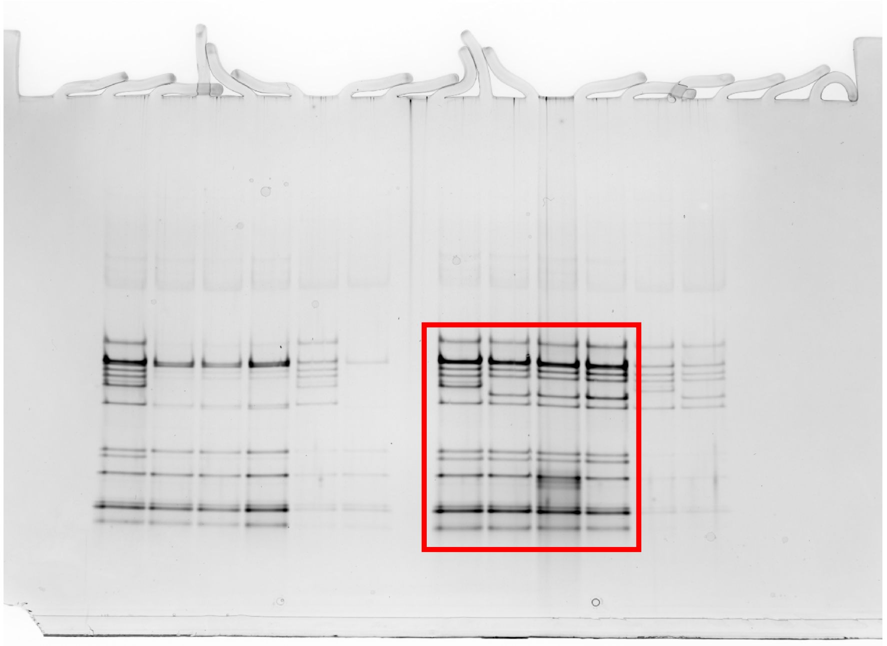

Supp. Fig. 2e

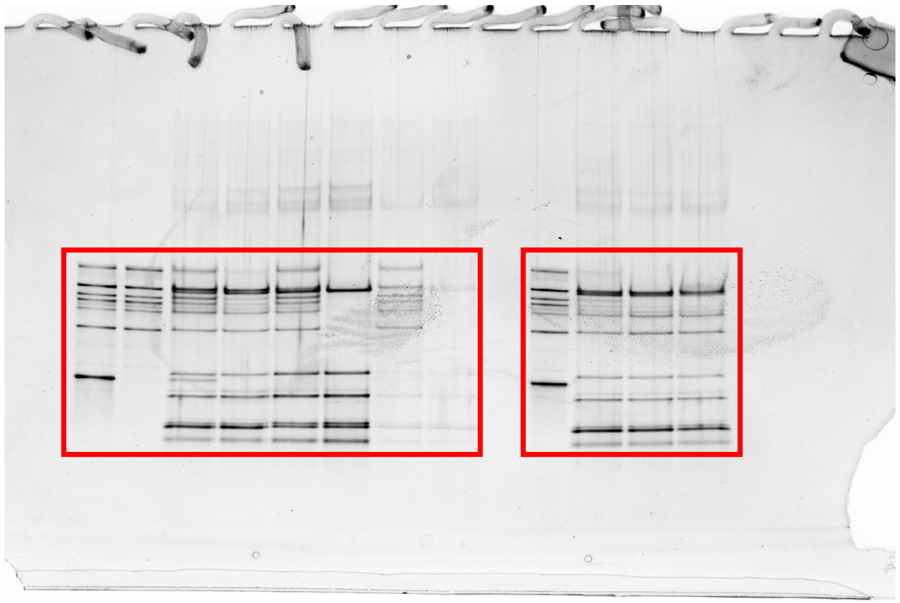

Supp. Fig. 3b

Supp. Fig. 3a

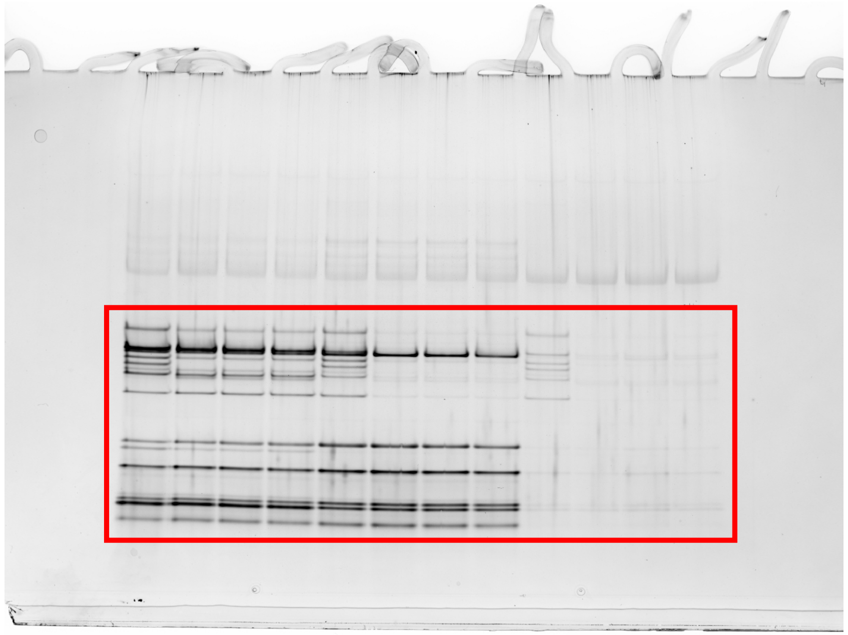

Supp. Fig. 4a

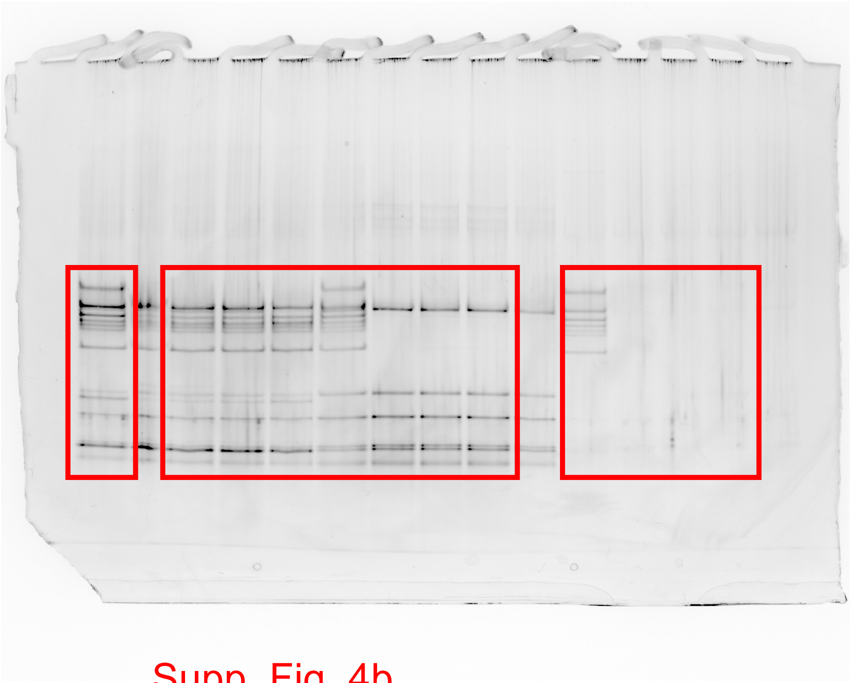

Supp. Fig. 4b

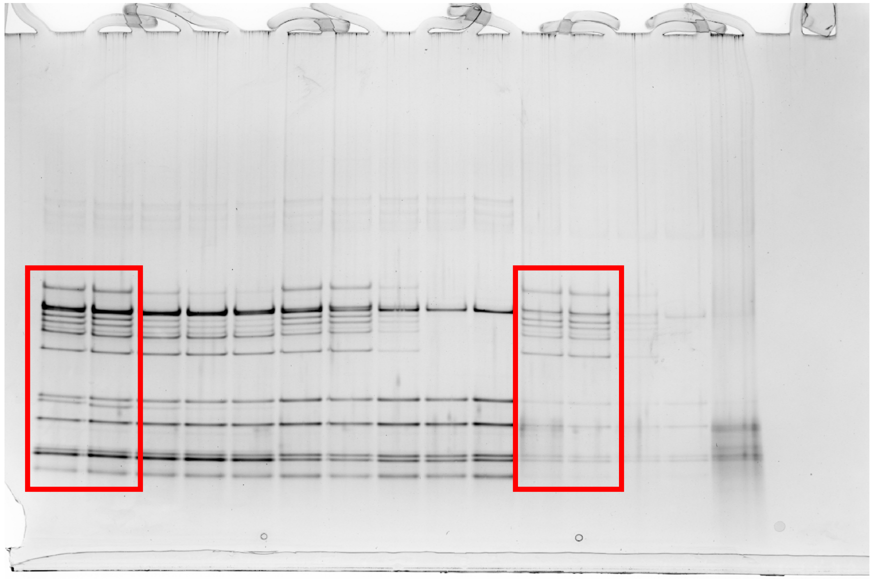

Supp. Fig. 6a

Supp. Fig. 6b

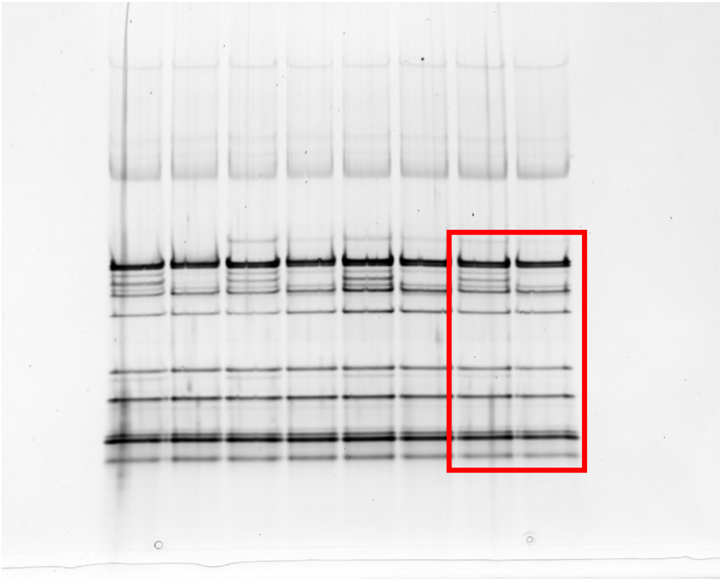

Supp. Fig. 6c
